# Mutation of *CRYAB* encoding a conserved mitochondrial chaperone and antiapoptotic protein causes hereditary optic atrophy

**DOI:** 10.1172/jci.insight.182209

**Published:** 2024-11-19

**Authors:** Chenghui Wang, Liyao Zhang, Zhipeng Nie, Min Liang, Hanqing Liu, Qiuzi Yi, Chunyan Wang, Cheng Ai, Juanjuan Zhang, Yinglong Gao, Yanchun Ji, Min-Xin Guan

**Affiliations:** 1 Center for Mitochondrial Biomedicine and Department of Ophthalmology, the Fourth Affiliated Hospital,; 2 Department of Genetics, and; 3 Center for Genetic Medicine, Zhejiang University International Institute of Medicine, Yiwu, Zhejiang, China.; 4 School of Ophthalmology and Optometry and Eye Hospital, Wenzhou Medical University, Wenzhou, Zhejiang, China.; 5 Division of Medical Genetics and Genomics, The Children’s Hospital, Zhejiang University School of Medicine and National Clinical Research Center for Child Health, Hangzhou, Zhejiang, China.; 6 Joint Institute of Genetics and Genomic Medicine between Zhejiang University and University of Toronto, Zhejiang University, Hangzhou, Zhejiang, China.

**Keywords:** Genetics, Ophthalmology, Apoptosis, Bioenergetics, Mitochondria

## Abstract

The degeneration of retinal ganglion cells (RGC) due to mitochondrial dysfunctions manifests optic neuropathy. However, the molecular components of RGC linked to optic neuropathy manifestations remain largely unknown. Here, we identified a potentially novel optic atrophy-causative CRYAB gene encoding a highly conserved major lens protein acting as mitochondrial chaperone and possessing antiapoptotic activities. The heterozygous CRYAB mutation (c.313G>A, p. Glu105Lys) was cosegregated with autosomal dominant inheritance of optic atrophy in 3 Chinese families. The p.E105K mutation altered the structure and function of CRYAB, including decreased stability, reduced formation of oligomers, and decreased chaperone activity. Coimmunoprecipitation indicated that the p.E105K mutation reduced the interaction of CRYAB with apoptosis-associated cytochrome *c* and voltage-dependent anion channel protein. The cell lines carrying the p.E105K mutation displayed promotion of apoptosis and defective assembly, stability, and activities of oxidative phosphorylation system as well as imbalance of mitochondrial dynamics. Involvement of CRYAB in optic atrophy was confirmed by phenotypic evaluations of Cryab^p.E105K^-knockin mice. These mutant mice exhibited ocular lesions that included alteration of intraretinal layers, degeneration of RGCs, photoreceptor deficits, and abnormal retinal vasculature. Furthermore, Cryab-deficient mice displayed elevated apoptosis and mitochondrial dysfunctions. Our findings provide insight of pathophysiology of optic atrophy arising from RGC degeneration caused by CRYAB deficiency–induced elevated apoptosis and mitochondrial dysfunctions.

## Abstract 2

CRYAB encoding a highly conserved protein acting as mitochondrial chaperone and possessing anti-apoptotic activities is the molecular component of retinal ganglion cells, which its dysfunctions led to defects in ganglion neurons and caused optic atrophy.

## Graphical abstract

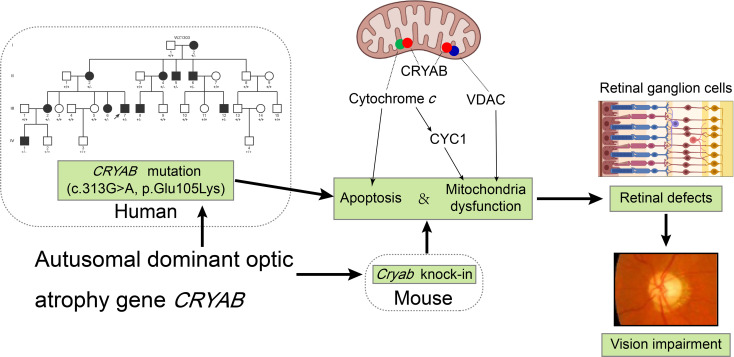

## Introduction

Mitochondria are the eukaryotic cell organelles that are responsible for producing ATP through the oxidative phosphorylation system (OXPHOS) (1). The retina, as one of highest energy-consuming organs, is vulnerable to mitochondrial dysfunction (2–4). In particular, the pigmented/photoreceptor layers and the ganglion cell/nerve fiber layers in the retina are exquisitely sensitive to mitochondrial dysfunctions. The degeneration of photoreceptors resulted in retinal dystrophy/retinitis pigmentosa (5, 6), while the degeneration of retinal ganglion cells resulted in optic neuropathy, including autosomal dominant optic atrophy (ADOA) and Leber’s hereditary optic neuropathy (LHON) (7–12). LHON is the most common maternally inherited eye disorder, characterized by central visual loss in young adults, due to mitochondrial DNA mutations (6–15). ADOA is characterized by progressive degeneration of retinal ganglion cells and their axons, causing insidious visual loss in both eyes, usually beginning in childhood and progressively worsening over time (16–19). The majority of ADOA cases worldwide have been associated with heterozygous mutations in OPA1 encoding mitochondrial dynamin-like GTPase that is involved in mitochondrial membrane biogenesis and stabilization of membrane integrity (20–22). Other ADOA-associated genes include AFG3L2 and SPG7 encoding mitochondrial AAA proteases, ACO2 coding for a matrix Krebs cycle enzyme, OPA3 encoding an integral protein of mitochondrial outer membrane, and SSBP1 coding for mitochondrial single-stranded DNA binding protein (23–28). However, molecular components of retinal ganglion cells linked to ADOA manifestations remain largely unknown.

Our recent studies demonstrated that LHON-associated mtDNA mutations including ND1 3460G>A, ND6 14484T>C, and ND4 11778G>A mutations only accounted for 48% cases of a large cohort of 1,793 Chinese probands with optic atrophy (13–15, 29–30). By whole exome sequencing of members among Chinese families lacking these LHON-associated mtDNA mutations, we identified a potentially novel heterozygous mutation (c.313G>A, p. Glu105Lys) in the αB-crystallin (CRYAB) gene encoding a major lens protein belonging to the small heat-shock family of proteins and possessing antiapoptotic activities in 3 families (31–35). The p.Glu105Lys (p.E105K) mutation resides at a highly conserved α-crystallin domain (ACD) (residues 66–148), which is involved in the oligomerization of proteins and regulating the function of the chaperone (36–38). In the present study, we investigated the effect of p.E105K mutation on structure, oligomerization, and chaperone function of αB-crystallin protein using molecular dynamics simulations and in vitro assays. Functional significance of the p.E105K mutation was further assessed for mitochondrial functions and apoptosis through the use of lymphoblastoid mutant and control cell lines derived from members of the Chinese family and CRYAB knockdown in dermal fibroblasts by shRNA. To investigate whether defects in CRYAB cause the phenotype of optic atrophy in vivo, we generated the Cryab p.E105K-knockin mice using the CRISPR/Cas9 system. These Cryab^E105K^ mice were further evaluated for the effect of Cryab mutation on the mitochondrial functions and apoptosis as well as retinal functions.

## Results

### Clinical presentation of 3 Han Chinese families with optic neuropathy.

**Figure 1:**
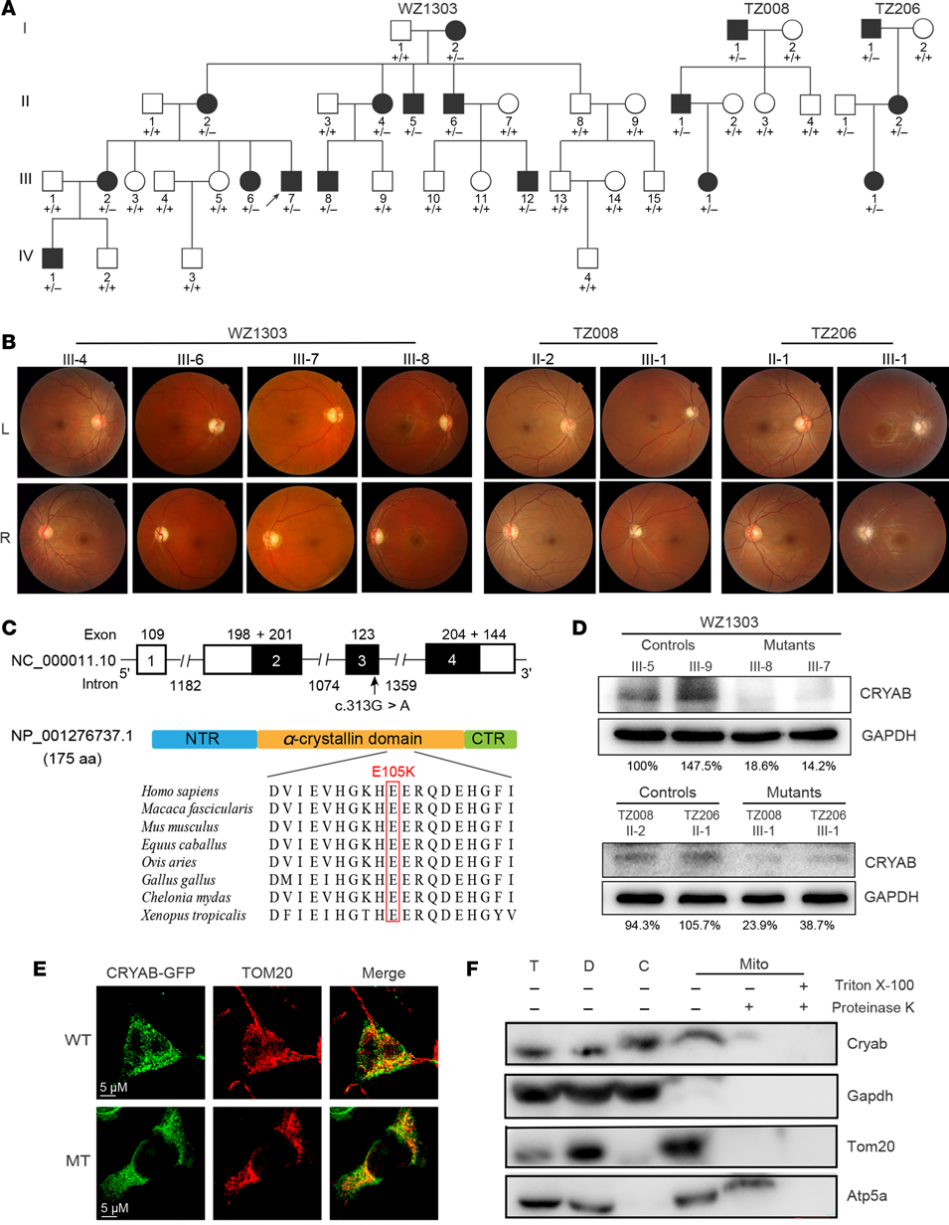
Identification of CRYAB p.E105K mutation. (**A**) Three Han Chinese pedigrees with optic nerve atrophy. Vision-impaired individuals are indicated by blackened symbols. Individuals harboring heterozygous (+/–) CRYAB (c.313G>A, p.Glu105Lys) mutation and WT (+/+) are indicated. (**B**) Fundus photographs from 8 (5 affected/3 control) members of 3 Chinese families. (**C**) Scheme for the structure of CRYAB and its products; multiple-sequence alignment of 7 homologs. (**D**) Western blot analysis of CRYAB in various lymphoblastoid cell lines. Representative of 3 independent experiments. (**E**) Subcellular localization of CRYAB by immunofluorescence in SH-SY5Y cells. CRYAB-GFP WT or MT (shown in green) and TOM20 (shown in red). Scale bar: 5 μm. (**F**) Subcellular localization of Cryab by Western blotting with Cryab, Tom20 (outer mitochondrial membrane), Atp5a (inner mitochondrial membrane), and Gapdh (cytosol). T, total cell lysate; D, debris; C, cytosol; Mito, mitochondria. Mitochondria isolated from C57 mice brain were treated with (+) or without (–) 1% Triton X-100 followed by proteinase K digestion.

Three genetically unrelated Han Chinese families (WZ1303, TZ008, and TZ206) lacking the known LHON-linked mtDNA mutations (Supplemental Table 1; supplemental material available online with this article; https://doi.org/10.1172/jci.insight.182209DS1) and pathogenic mutations in a targeted gene panel of 37 genes involved in hereditary optic neuropathies (27, 28) were used for this investigation. These pedigrees exhibited an autosomal dominate inheritance of optical neuropathy (Figure 1A). Seventeen (8 females and 9 males) of 42 members among these families exhibited bilateral optical neuropathy as the sole clinical symptom. Members of these families displayed relatively mild visual impairment, ranging from moderate to mild visual impairment. Three members suffered from acute vision loss, while vision impairment of the other 14 members developed from 1 to 4 weeks. The age-at-onset of visual impairment varied from 14 to 24 years, with the average of 18.9 years old (Figure 1A and Supplemental Table 2). As showed in Figure 1B, fundus examination showed that patients exhibited vascular tortuosity of the central retinal vessels, a circumpapillary telangiectatic microangiopathy, and swelling of the retinal nerve fiber layer. These suggested the unprecedented combination of dominant inheritance and LHON-like phenotype in these families (2, 9, 16, 17). Further ophthalmological examination failed to observe other eye disorders including cataracts in all family members. Comprehensive clinical examinations showed that these family members showed no other clinical abnormalities, such as cardiomyopathy, myopathy, progressive muscle weakness, diabetes, hearing impairment, or neurological disorders.

### Identification of CRYAB mutation by whole exome sequencing.

To identify new causative genes of optic neuropathy, we subjected 4 members (proband III-7, affected mother II-2, unaffected father II-1, and uncle II-8) of WZ1303 family to whole exome sequences. The overview of exome analysis was summarized in Supplemental Figure 1 and Supplemental Table 3. After removing annotated polymorphisms and a series of filtering for variants, we identified a heterozygous single exonic variant (c.313G>A, p.E105K) (chromosome 11q23.1) in exon 3 of the αB-crystallin (CRYAB) gene encoding a major structural protein present in the lens of vertebrate eyes (31–33). The c.313G>A mutation changed a highly conserved 105 glutamic acid with lysine (p.E105K) at the ACD (residues 66–148) of CRYAB (Figure 1C). CRYAB is a highly conserved mitochondrial protein belonging to a small heat shock family and possessing antiapoptotic activities (Supplemental Figure 2) (33–38). We then performed the Sanger sequence analysis of DNA fragments spanning all exons and their flanking sequences of CRYAB among 15 affected patients and 23 unaffected members of these families (Supplemental Table 4). This p.E105K mutation was present in all 15 affected patients but not in 23 unaffected members in these Chinese families. No other sequence change in this gene was detected among these individuals. We further analyzed the presence of c.313G>A mutation in a cohort of 757 genetically unrelated probands with optic neuropathy and 316 unrelated non–vision-impaired individuals by Sanger sequencing. The c.313G>A mutation was absent among these unrelated non–vision-impaired and vision-impaired participants. The cosegregation of optic neuropathy with the presence of heterozygous c.313G>A mutation in 10 males and 7 females suggested that the CRYAB c.313G>A mutation is a rare ADOA causative allele.

To investigate the effect of the p.E105K mutation on CRYAB, we examined the level of CRYAB by Western blot analysis using mutant and control lymphoblastoid cell lines derived from members of WZ1303, TZ008, and TZ206 pedigrees. As shown in Figure 1D, the cell lines carrying the p.E105K mutation revealed marked reductions in the level of CRYAB, indicating deleterious effect of p.E105K mutation on CRYAB structure/function. To examine whether the p.E105K mutation affected the subcellular localization of CRYAB, pEGFP-N1-CRYAB expressing the WT and mutant (MT) GFP fusion proteins were transfected into the SH-SY5Y cell line, respectively. As shown in Figure 1E, both C-terminal GFP-tagged WT or MT CRYAB displayed the overlap with mitochondrial protein TOM20, indicating that the p.E105K mutation did not change the mitochondrial localization of CRYAB. Locations of CRYAB in the compartments of mitochondria were further examined by fractioning the mouse brain cells into mitochondrial and cytosolic fractions and Western blot analysis, along with Cryab, Tom20 (outer mitochondrial membrane protein), Atp5a (inner mitochondrial membrane protein), and Gapdh (cytosolic protein), respectively (39). As shown in Figure 1F, Cryab was enriched in outer mitochondrial membrane but also present in the inner mitochondrial membrane.

### Altered structure and function of CRYAB.

**Figure 2:**
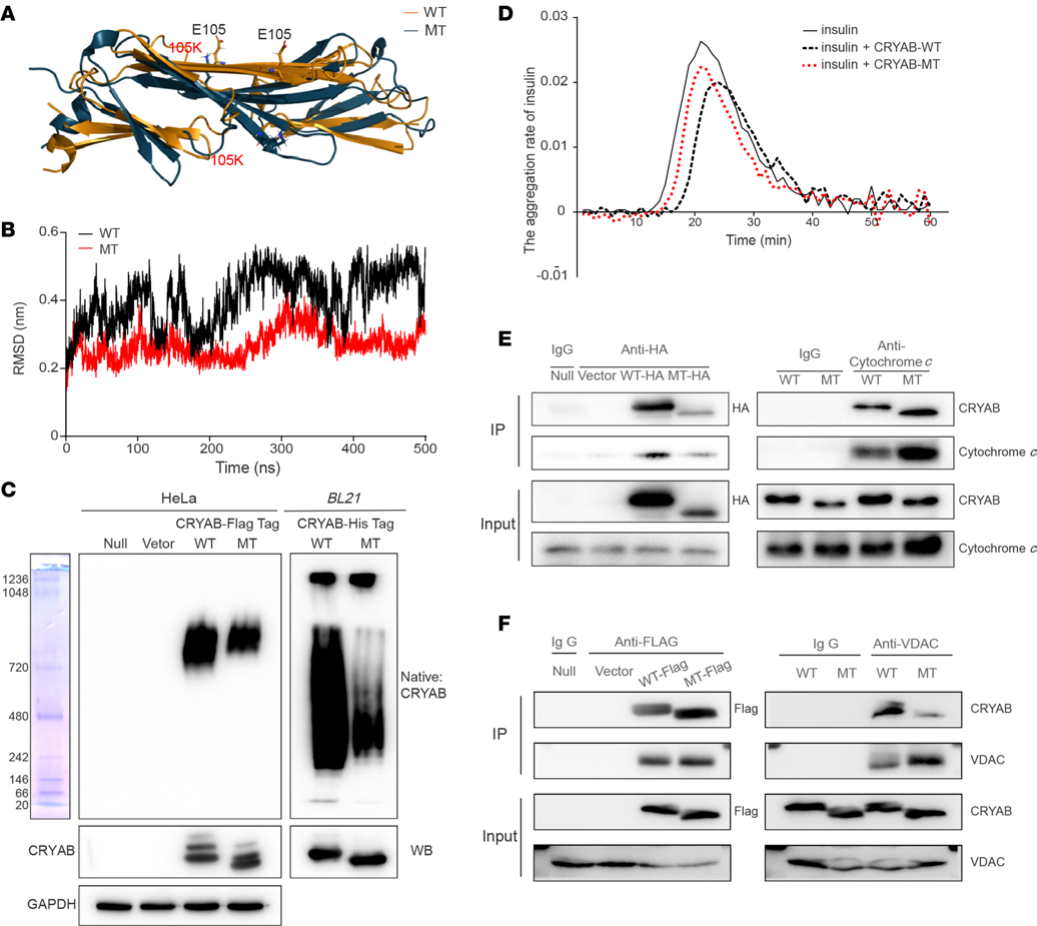
In vitro analysis of structure and function of WT and MT CRYAB. (**A**) Schematic model for the α-crystallin domain (ACD) of CRYAB homodimer (PDB ID: 2klr) for WT (tawny) and MT (blue) contains chain A and chain B after MD simulations of 500 ns. The MT site was indicated. (**B**) Time evolution of the root mean square deviation (RMSD) values of all α-carbon (α-Cα) atoms for the WT (black line) and MT (red line) proteins. (**C**) Analysis for the oligomer formation of CRYAB. Both WT and MT proteins, labeled by Flag/His Tag, were expressed and purified from HeLa and BL21, electrophoresed through a blue native gel, and electroblotted and hybridized with antibodies specific for Flag-Tag, His-Tag, CRYAB, and GAPDH. (**D**) The chaperone activities of MT and WT CRYAB with DTT-induced denatured insulin. Insulin (0.4 mg/mL in 50 mM phosphate buffer [pH 7.4]) was reduced with 20 mM DTT, and the aggregation of the insulin CRYAB was monitored by measuring the apparent absorption at 360 nm. The aggregation rates were calculated. (**E** and **F**) Reduced interactions of CRYAB with cytochrome *c* (**E**) or VDAC (**F**). HEK293T cells, transiently expressing with WT, MT CRYAB-HA, or CRYAB-FLAG and empty vector, were solubilized with a lysis buffer. Lysate proteins and immunoprecipitates were immunoprecipitated with immunocapture buffer (IgG) (left), cytochrome *c* or VDAC antibody (right). Immunoprecipitates were then analyzed by SDS-PAGE and hybridized with anti-CRYAB and anti–cytochrome *c* or with anti-VDAC antibodies. Representative of 3 independent experiments.

We examined the effect of p.E105K mutation on the structure and function of CRYAB using in vitro assays. The ACD is the central element for the ability of CRYAB to assemble into oligomers (35, 36). First, we carried out the molecular dynamics simulation to assess the effects of p.E105K mutation on the structure and function of CRYAB. Based on the rational initial structure, both WT and MT ACD were evaluated by 500 ns all-atom molecular dynamics simulation, followed by the equilibrated system. As shown in Figure 2A, the p.E105K mutation perturbed the homodimer structure of ACD of αB-crystallin protein. As shown in Figure 2B, root mean square deviation (RMSD) curve of mutated protein fluctuated much less than that of WT counterpart, suggesting that the mutated protein exhibited less flexibility than its WT counterpart.

We then examined if the p.E105K mutation impaired the oligomer formation by expressing WT and MT CRYAB with C-terminal Flag-tagged in HeLa cells and with C-terminal His-tagged in bacterial cells. Native α-crystallin from vertebrate eye lens displayed polydispersity with a broad molecular mass distribution (300–1,000 kDa) (34, 35). As shown in Figure 2C, electrophoretic patterns showed that the p.E105K mutation led to decreased formation of oligomers and changed the conformation, evidenced by faster migration of MT CRYAB than those of WT molecules.

We then investigated the effect of p.E105K mutation on its chaperone function. The chaperone-like activities of recombinant His-tagged human WT and MT CRYAB were evaluated by measuring the ability to suppress DTT-induced aggregation of model substrate recombinant human insulin (38). As shown in Figure 2D, MT CRYAB revealed a faster aggregation rate and earlier starting time of aggregation, as compared with WT counterpart, indicating that the mutation reduced chaperone activity of this protein.

The previous studies show that CRYAB interacts with cytochrome *c* and voltage-dependent anion channel protein (VDAC) to protect it against oxidative stress (37, 40). The effects of p.E105K mutation on interaction between CRYAB and cytochrome *c* or VDAC were examined by the immunoprecipitation assays. As shown in Figure 2, E and F, the relative levels of CRYAB versus cytochrome *c* or VDAC in MT protein revealed marked reductions, indicating weaker interactions between MT CRYAB with cytochrome *c* or VDAC than those in WT CRYAB. These suggested the effect of p.E105K mutation on apoptosis or mitochondrial function.

### Promoted apoptotic process.

**Figure 3:**
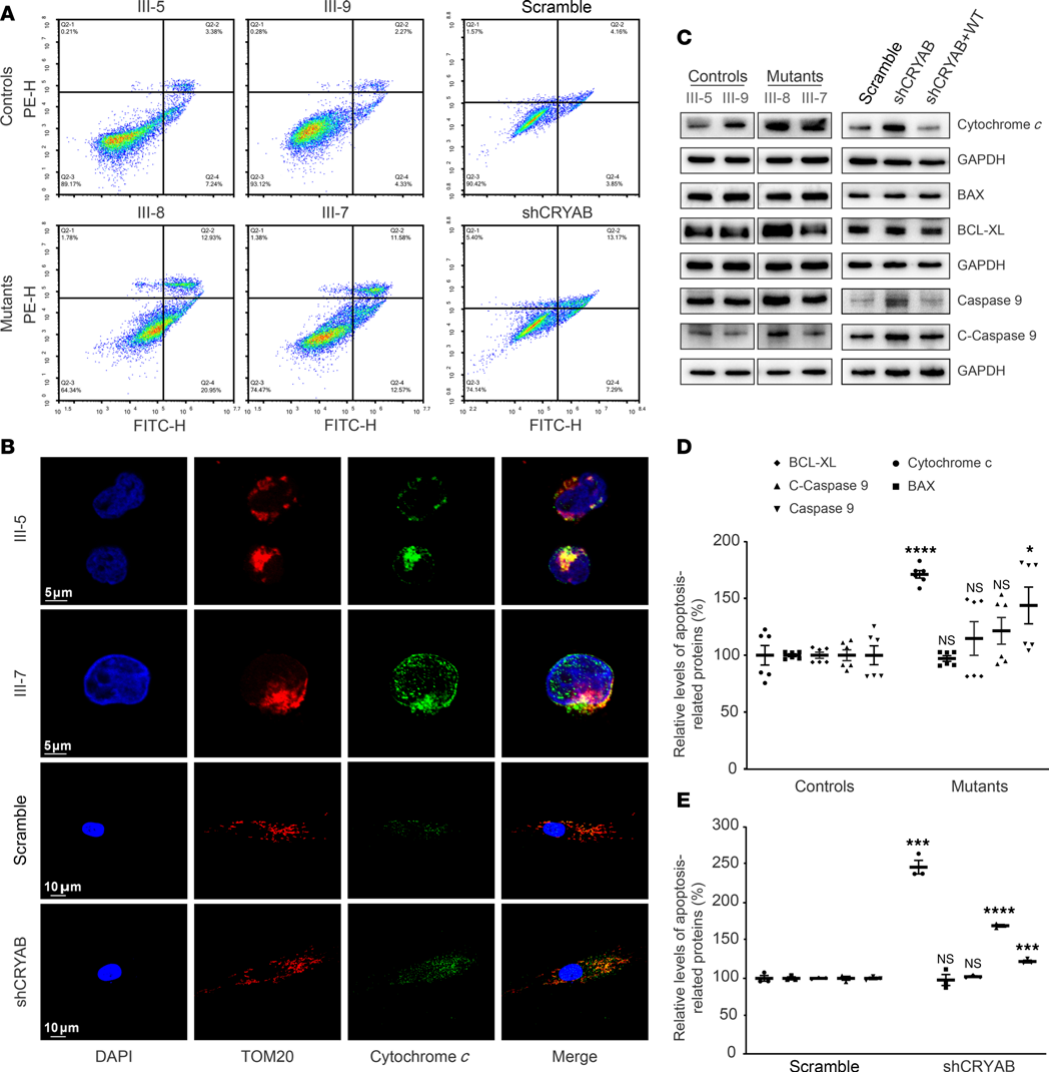
Apoptosis assays. (**A**) Annexin V/PI apoptosis assay by flow cytometry. Cells were harvested and stained with annexin V and 1 μL of propidium iodide. The percentage of annexin V^+^ cells were then assessed. (**B**) Immunofluorescence analysis. The distributions of cytochrome *c* were visualized by immunofluorescence labeling with TOM20 antibody conjugated to Alexa Fluor 594 (red) and cytochrome *c* antibody conjugated to Alexa Fluor 488 (green) analyzed by confocal microscopy. DAPI-stained nuclei were identified by their blue fluorescence. (**C**) Western blot analysis. In total, 20 μg of total cellular proteins from various cell lines were electrophoresed, electroblotted, and hybridized with several apoptosis-associated protein antibodies: cytochrome *c*, BAX, BCL-XL, or uncleaved/cleaved caspases-9, with GAPDH as a loading control. (**D** and **E**) Quantification of apoptosis-associated proteins: cytochrome *c*, BAX, BCL-XL, and uncleaved and cleaved caspase-9. Data are as shown as mean ± SEM of triplicates. *P < 0.05; ***P < 0.001; ****P < 0.0001, by Student’s *t* test, show the differences between MT and control cell lines.

We evaluated if the p.E105K mutation impaired the apoptotic process using 4 lymphoblastoid cell lines derived from affected members and married-in controls of the Chinese family and CRYAB-knockdown cell line by annexin V/PI–based flow cytometry, immunocytostaining, and Western blot analyses. As shown in Figure 3A, 2 MT cell lines bearing the p.E105K mutation and CRYAB-knockdown cell line displayed marked increases in the average rations of annexin V^+^ cells, as compared with those in the control cell lines, respectively. We then examined the apoptotic states of various cell lines by using immunocytostaining assays that show immunofluorescence patterns of double labeled cells with antibodies specific for cytochrome *c* and TOM20. As shown in Figure 3B, there were higher levels of cytosolic cytochrome *c* in MT cell lines and knockdown cell line than those in the control cell lines. The effect of p.E105K mutation on the apoptotic process was further analyzed with Western blot analysis. As shown in Figure 3, C–E, the levels of cytochrome *c* in the MT cell lines bearing the p.E105K mutation and knockdown cell line increased 71% and 147%, relative to the mean values in the control cell lines, respectively. Furthermore, we measured the levels of apoptosis-related proteins: BAX, BCL-XL, uncleaved/cleaved caspase-9 in MT, and control cell lines by Western blot analysis. As shown in Figure 3, C–E, MT cell lines bearing the p.E105K mutation and knockdown cell line exhibited elevated levels of uncleaved/cleaved caspase-9, as compared with control cell lines, respectively, while the levels of BAX and BCL-XL in the MT cell lines were comparable with those in control cell lines. These results indicate that the p.E105K mutation promoted apoptotic process.

### Defective stability, assembly, and activity of OXPHOS.

**Figure 4:**
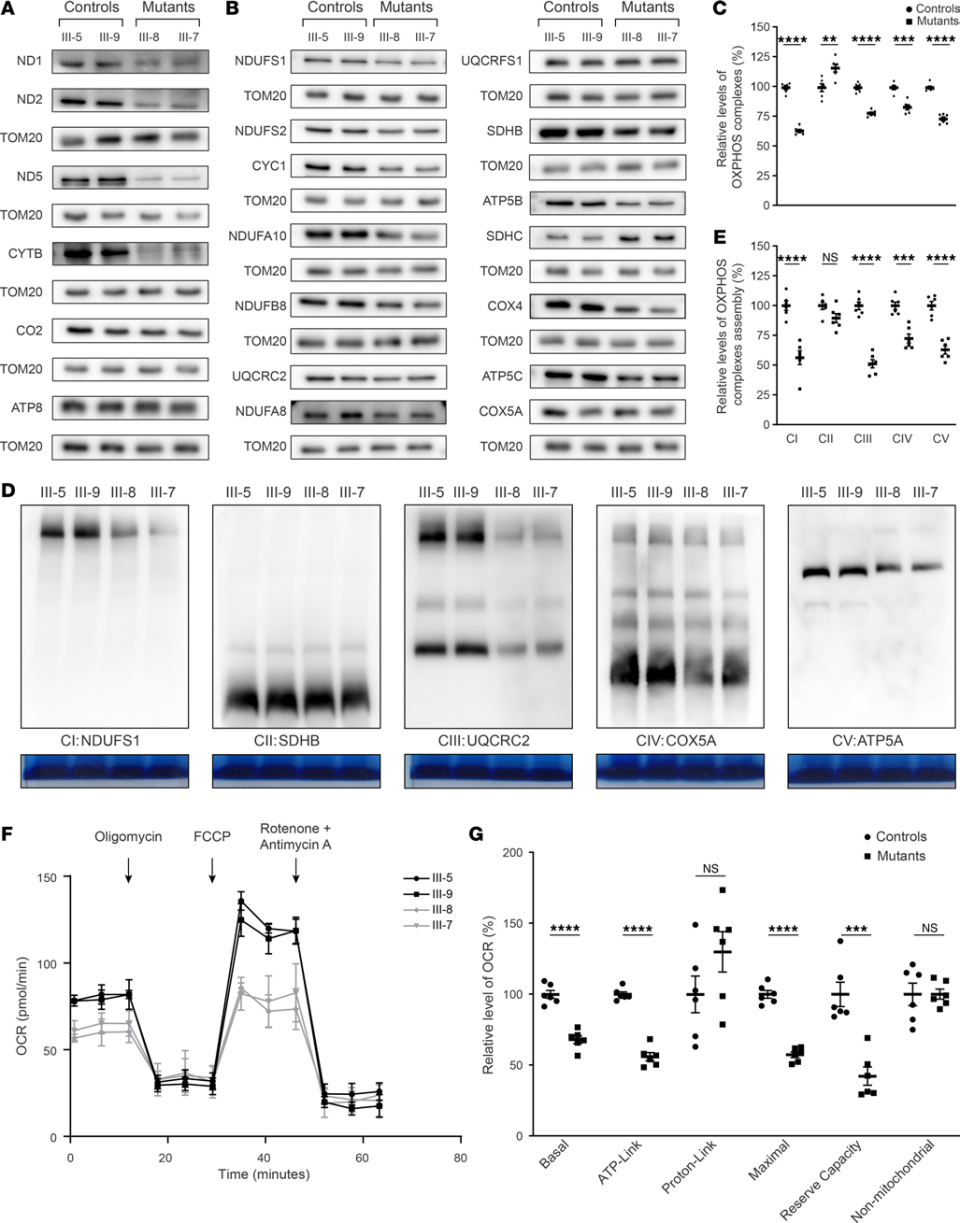
Analysis of mitochondrial functions. (**A** and **B**) Twenty micrograms of total cellular proteins from various cell lines were electrophoresed through a denaturing polyacrylamide gel, electroblotted, and hybridized with antibodies for 20 subunits of OXPHOS (6 encoded by mtDNA and 14 encoded by nuclear genes) and TOM20 as a loading control. (**C**) Average levels of subunits from each complex of OXPHOS (8 of complex I, 2 of II, 4 of III, 3 of IV, and 3 of V). (**D**) The steady-state levels of 5 OXPHOS complexes by BN-gel electrophoresis. A total of 30 μg of mitochondrial proteins from various cell lines were electrophoresed through a BN gel, electroblotted, and hybridized with antibodies specific for subunits of 5 OXPHOS complexes (NDUFS1 antibody for complex I, SDHB antibody for complex II, UQCRC2 antibody for complex III, COX5A antibody for complex IV, and ATP5A antibody for complex V), and Coomassie staining was used as a loading control. (**E**) Quantification of levels in the complexes I, II, III, IV, and V in MT and WT cell lines. (**F** and **G**) Seahorse analysis in various lymphoblastoid cell lines. (**F**) An analysis of O_2_ consumption in the various cell lines using different inhibitors. (**G**) Graphs presented the basal OCR, ATP-linked OCR, proton leak OCR, and maximal OCR in cell lines. Data are shown as mean ± SEM of triplicates. Student’s *t* test. **P < 0.01; ***P < 0.001; ****P < 0.0001.

We assessed the effect of CRYAB mutation on the mitochondrial function, we carried out the Western blotting analysis to examine the levels in 20 subunits of OXPHOS complexes in 2 MT and 2 control cell lines using TOM20 as a loading control. These subunits included 6 mtDNA encoding polypeptides (ND1, ND2, ND5, CYTB, CO2, and ATP8) and 14 nucleus encoding proteins: NDUFS1, NDUFS2, NDUFA8, NDUFA10, and NDUFB8 (subunits of complex I); SDHB and SDHC (subunits of succinate dehydrogenase [complex II]); CYC1, UQCRFS1, and UQCRC2 (subunits of ubiquinol-cytochrome *c* reductase [complex III]); COX4 and COX5A (subunits of complex IV); and ATP5B and ATP5C (subunits of H^+^-ATPase [complex V]) (1). As shown in Figure 4, A and B, various decreases in the levels of 20 mitochondrial proteins (but not of SDHC, UQCRFS1, CO2, COX5A, or ATP8) were observed in the MT cell lines, as compared with the WT cells. As shown in Supplemental Figure 3A, the levels of ND1, ND2, ND5, CYTB, CO2, and ATP8 in the MT cell lines were 70%, 54%, 31%, 33%, 97%, and 98% relative to the mean values measured in the WT cells. As shown in the Supplemental Figure 3B, the levels of NDUFS1, NDUFS2, NDUFA8, NDUFA10, NDUFB8, SDHB, SDHC, CYC1, UQCRFS1, UQCRC2, COX4, COX5A, ATP5B, and ATP5C in the MT cell lines were 66%, 76%, 74%, 60%, 76%, 66%, 167%, 62%, 106%, 85%, 56%, 98%, 55%, and 67%, relative to the mean values measured in the WT cells, respectively. Notably, the average levels in the subunits of complexes I, II, III, IV, and V in the MT cell lines were 63%, 117%, 78%, 83%, and 74% of average values measured in the WT cells, respectively (Figure 4C).

We further analyzed the consequence of CRYAB mutation on the oxidative phosphorylation machinery. Mitochondrial membrane proteins isolated from MT and control cell lines were separated by blue native–PAGE (BN-PAGE), electroblotting and hybridizing with human NDUFS1, SDHB, UQCRC2, COX5A, and ATP5A antibodies (41, 42). As illustrated in Figure 4D, the MT cell lines displayed the instability of complexes I, III, IV, and V. As shown in Figure 4E, the average levels of complexes I, II, III, IV, and V in the MT cell lines were 56%, 90%, 51%, 72%, and 63% of those average values in control cell lines, respectively. Furthermore, the CRYAB-knockdown cell line exhibited defective stability and assembly of OXPHOS, and overexpression of CRYAB cDNAs in the CRYAB-knockdown cell line restored these defects (Supplemental Figure 4).

To further assess if the p.E105K mutation impaired mitochondrial function, we measured the OCR of various cell lines via extracellular flux analyzer (43). As shown in Figure 4, F and G, the basal OCR in the cell lines carrying the p.E105K mutations was 68% of the mean value measured in the control cell lines. To investigate which of the enzyme complexes of the respiratory chain were affected in the MT cell lines, oligomycin (to inhibit the ATP synthase), FCCP (to uncouple the mitochondrial inner membrane and allow for maximum electron flux through the electron transport chain), rotenone (to inhibit complex I), and antimycin A (to inhibit complex III) were added sequentially while measuring OCR. The difference between the basal OCR and the drug-insensitive OCR yields the amount of ATP-linked OCR, proton leak OCR, maximal OCR, reserve capacity, and nonmitochondrial OCR. As shown in Figure 4G, the ATP-linked OCR, proton leak OCR, maximal OCR, reserve capacity, and nonmitochondrial OCR in MT cell lines were 56%, 130%, 57%, 42%, and 100%, relative to the mean value measured in the control cell lines, respectively.

We then evaluated the effect of CRYAB mutation on mitochondrial dynamics using CRYAB-knockdown cell lines by immunofluorescence and Western blot analysis. As shown in Supplemental Figure 5A, CRYAB-knockdown cells exhibited abnormal mitochondrial morphology, as compared with those in scramble cells. The imbalance of mitochondrial dynamics was further supported by increasing levels of fusion-related proteins OPA1 and MFN1 and reduced levels of fission-related proteins DRP1 and FIS1 (Supplemental Figure 5B) (44). The defects may reflect on mtDNA copy numbers, evidenced by 36% increase of copy numbers of mtDNA in CRYAB-knockdown cells (Supplemental Figure 5C).

### Cryab^E105K^ mice exhibited the retinal defects.

**Figure 5:**
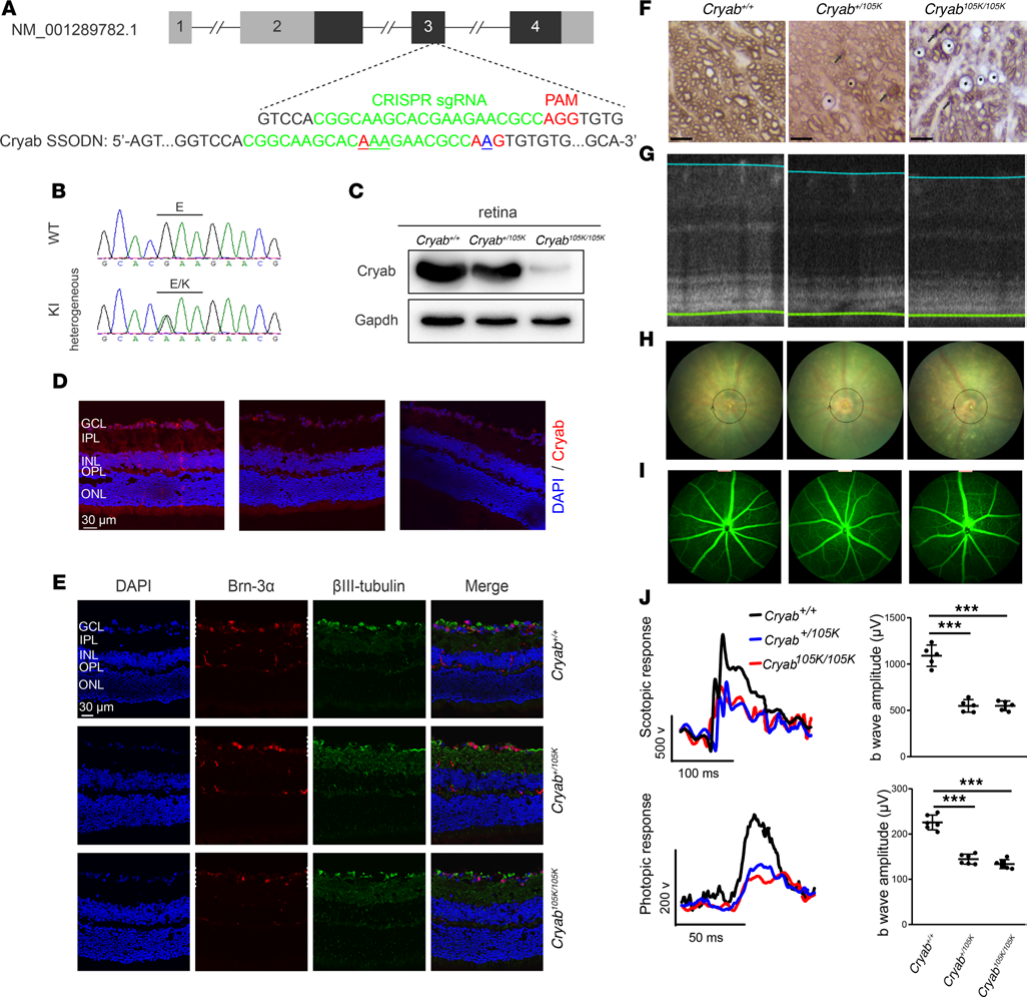
Retinal deficiencies in Cryab^E105K^ mice. (**A**) Schema for the generation of CRYAB-knockin mice (C57BL/6J) using the CRISPR/Cas9 system. (**B**) Sequence chromatograms of Cryab gene. Sanger sequencing of WT and Cryab^+/105K^ mice. (**C**) Levels of Cryab in the retinas from Cryab^+/+^, Cryab^+/105K^, and Cryab^105K/105K^ mice at 8 weeks of age. (**D**) Cryab expression in the retinas of Cryab^+/+^, Cryab^+/105K^, and Cryab^105K/105K^ mice by immunolabeling analysis. GCL, ganglion cell layer; IPL, inner plexiform layer; INL, inner nuclear layer; OPL, outer plexiform layer; ONL, outer nuclear layer. Scale bar: 30 μm. (**E**) RGCs were stained with Brn-3a (green), β-III-tubulin (red), and DAPI (blue). Scale bar: 30 μm. (**F**) Ultrastructural analysis of RGC axons showing swelling and loss in Cryab^+/105K^ and Cryab^105K/105K^ mice. Swollen axons with thin myelin (asterisks) and degenerated axons with myelin clumping (arrow) were seen adjacent to normal caliber axons. Scale bar: 5 μm. (**G**) Representative optic coherence tomography of the retina in Cryab^+/+^, Cryab^+/105K^, and Cryab^105K/105K^ mice. The circumpapillary retinal thickness was measured by OCT circle scan. (**H**) Fundus photograph eyes in Cryab^+/+^, Cryab^+/105K^, and Cryab^105K/105K^ mice with OCT circle scan sites (black circles). (**I**) Fluorescence angiography of eyes in Cryab^+/+^, Cryab^+/105K^, and Cryab^105K/105K^ mice. (**J**) Analysis of ffERG for Cryab^+/+^ (*n* = 6), Cryab^+/105K^ (*n* = 6), and Cryab^105K/105K^ (*n* = 6) mice. By dark adaptation for a night, mice were analyzed for scotopic response and then photopic response. Data are shown as mean ± SEM of triplicates. ***P < 0.001, by 1-way ANOVA followed by Bonferroni’s post hoc test.

To investigate whether defects in Cryab cause the dysfunction of visual systems in vivo, we studied the Cryab^E105K^ mice produced by the CRISPR/Cas9 system (Figure 5, A and B). Both Cryab^+/105K^ and Cryab^105K/105K^ mice were viable and developed the retinal deficiency at the 8 weeks old. However, both heterozygous and homozygous MT mice did not develop myopathy and cataracts, unlike the phenotype of knockin mouse model for the R120G mutation (45). Both Western blot and IHC data show that Cryab was reduced in the retinas of Cryab^+/105K^ and Cryab^105K/105K^ mice (Figure 5, C and D). The Cryab was ubiquitously expressed in various layers of retina in mice (Figure 5D), consistent with the expression patterns of Cryab in human retina (46). Ablation of Cryab caused various reductions of Cryab in various layers of retina, especially pronounced decreases in the retinal ganglion cell layer (GCL) (Figure 5D). The degeneration of RGCs in the Cryab^+/105K^, Cryab^105K/105K^, and WT mice was further evaluated by immunostaining GCL with RGC markers Brn3a and β-III-tubulin antibodies as well as DAPI to show nuclei (47). Strikingly, the RGCs (Brn3a^+^ staining) in the retinal ganglion layers of Cryab^+/105K^ and Cryab^105K/105K^ mice were significantly reduced, as compared with WT littermates (Figure 5E and Supplemental Figure 6A). Ultrastructural analysis of RGC axons in the optic nerve cross sections showed axonal swelling and degeneration in the optic nerves of both Cryab^+/105K^ and Cryab^105K/105K^ eyes (Figure 5F). The numbers of axons in the optic nerves of Cryab^+/105K^ and Cryab^105K/105K^ eyes were decreased 20% and 24%, as compared with WT mice, respectively (Supplemental Figure 6B). Furthermore, we performed IHC of mouse retina using rhodopsin^+^ for rod photoreceptor, Calb1^+^ for horizontal cells, Pkc-α^+^ for bipolar cells, and vimentin^+^ for Müller cells. As shown in Supplemental Figure 6C, there were no significant differences of these stainings in retina between MT and WT littermates. These results suggest that the Cryab mutation only caused the degeneration of RGCs but did not affect bipolar cells, Müller cells, and rods in the mouse retinas.

We further assessed the effect of Cryab mutation on vision function. Optic coherence tomography showed significantly decreased thickness in all intraretinal layers in the Cryab^+/105K^ and Cryab^105K/105K^ mice at 8 weeks of age (Figure 5G and Supplemental Figure 6D). Both fundus and fluorescein angiography analyses revealed ocular lesions and abnormal retinal vasculatures that were tortuous and dilated, with thinner vessels in the Cryab^+/E105K^ and Cryab^E105K/E105K^ mice, as compared with those in WT mice (Figure 5, H and I). Retinal functions in the Cryab^+/105K^, Cryab^105K/105K^, and WT mice were then assessed by full-field electroretinogram (ffERG), focusing on photoreceptor deficits. As shown in Figure 5J, the amplitude of b-wave for scotopic (rod) responses and photopic (cone) responses of Cryab^+/105K^ and Cryab^105K/105K^ mouse eyes at age of 8 weeks were declined 50% and 52%, as compared with WT mice, respectively, while the amplitude of b-wave for photopic (cone) responses of Cryab^+/105K^ and Cryab^105K/105K^ mouse eyes at age of 8 weeks were decreased, 36% and 41%, as compared with WT mice, respectively. These results suggested that the photoreceptor deficits or the ERG phenotypes were the specific manifestations of RGCs, caused by the Cryab mutation.

### Cryab^E105K^ mice revealed the impairment of apoptosis and OXPHOS.

**Figure 6:**
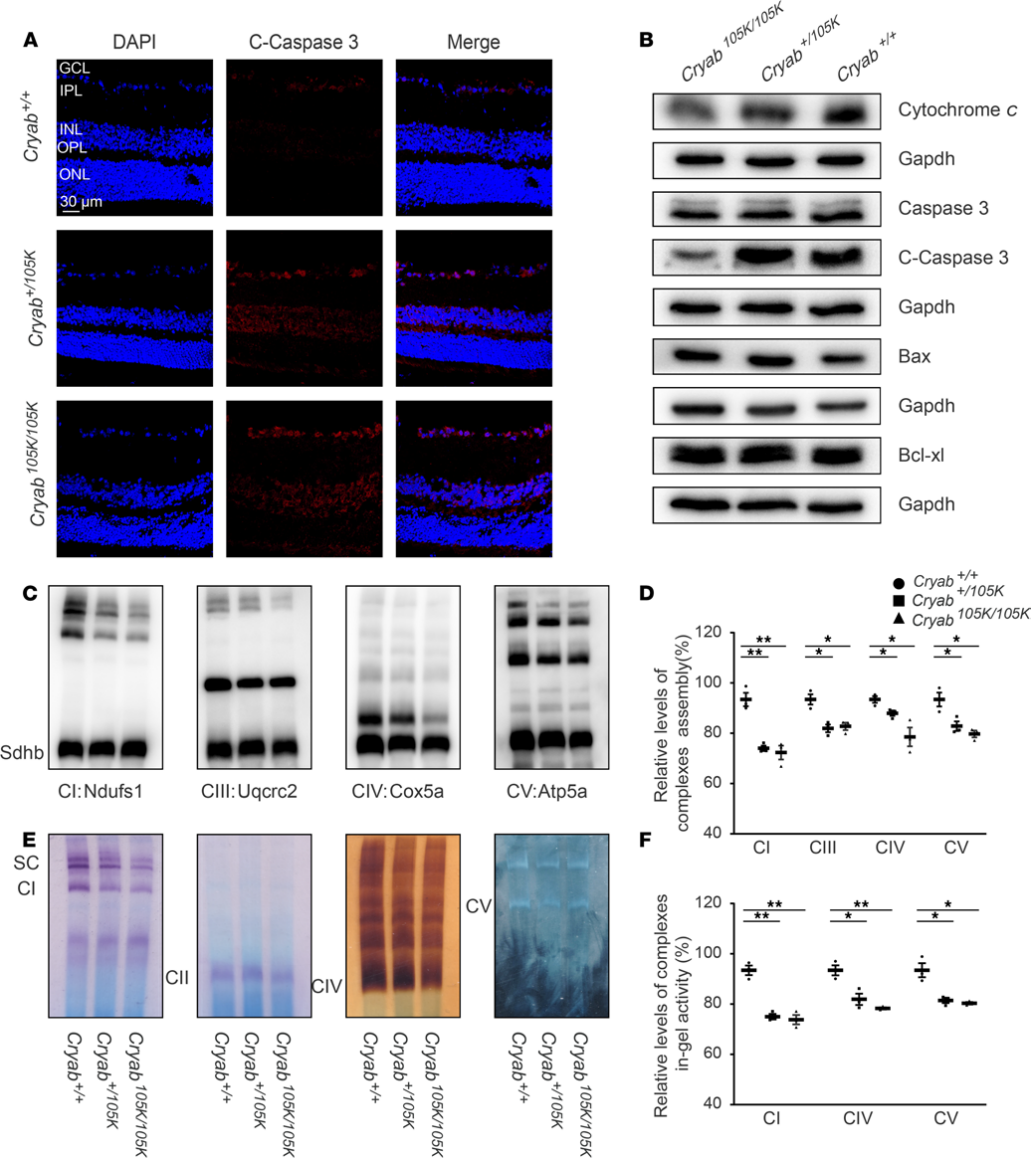
Assessment of apoptosis and OXPHOS activities. (**A**) Fluorescence analysis of cleaved caspase-3 protein level in mouse retina at 8 weeks of age. Cleaved caspase-3 was shown in red fluorescence, and DAPI-labeled nucleus was shown in blue fluorescence. (**B**) Western blot analysis. Proteins (20 μg) from mouse retinas were electrophoresed, electroblotted, and hybridized with several apoptosis-associated protein antibodies: cytochrome *c*, uncleaved/cleaved caspase-3, Bax and Bcl-xl, with GAPDH as a loading control. (**C**) The steady-state levels of 5 OXPHOS complexes by BN-PAGE. Mitochondrial proteins (30 μg) from MT and WT mice brains were electrophoresed through a BN gel, electroblotted, and hybridized with antibodies for Ndufs1, Sdhb, Uqcrc2, Cox5a, and Atp5a (subunits of complex I, II, III, IV, and V, respectively). (**D**) Quantification of relative levels of complexes assembly. (**E**) In-gel activities of complexes I, II, IV, and V. Mitochondrial proteins from MT and WT mice were used for BN gel, and the activities of complexes were measured in the presence of specific substrates, as described previously (42). (**F**) Average relative levels of complexes I, IV, and V content per cell were normalized to the average levels per cell of complex II in the MT and WT mice brains. Data are shown as mean ± SEM of triplicates. *P < 0.05; **P < 0.01, by 1-way ANOVA followed by Bonferroni’s post hoc test.

To test whether the Cryab deficiency promoted the apoptosis in Cryab^E105K^ mice, we measured the apoptotic state of MT and WT mice by immunofluorescence and Western blot analyses. The retinas at 8 weeks of age were stained with cleaved caspase-3 antibody and DAPI, to show nuclei. As shown in Figure 6A, the levels of cleaved caspase-3 were significantly elevated in the GCL of Cryab^+/105K^ and Cryab^105K/105K^ mice, as compared with control mice. The effect of the Cryab mutation on the apoptotic process was further evaluated with Western blot analysis. As shown in Figure 6B and Supplemental Figure 6E, the retinas of Cryab^+/105K^ and Cryab^105K/105K^ mice exhibited significantly increased levels of cytochrome *c* and cleaved caspase-3, respectively, as compared with Cryab^+/+^ mice. However, there were no significant differences in the average levels of uncleaved caspases-3, Bax, or Bcl-xl between MT and WT mice retina (Figure 6B). These results indicate that the defect of Cryab promoted apoptosis in retinas.

We examined the consequence of the Cryab deficiency on the assembly and activities of OXPHOS complexes. Mitochondrial membrane proteins isolated from MT and WT mouse brains were separated by BN-PAGE and Western blot analysis (41, 42). As shown in Figure 6, C and D, MT mouse brains exhibited aberrant assembly of supercomplexes, complexes I, III, IV, and V. As illustrated in Figure 6, E and F, the in-gel activities of complexes I, IV, and V were significantly decreased in the brains of MT mice, as compared with those in the brains of WT mice.

## Discussion

The major structural proteins of eyes linked to ADOA manifestations remain largely unknown. Using the whole exome sequencing approach, in combination with functional assays and animal disease model, we identified a potentially novel ADOA-causative gene CRYAB encoding a major lens protein acting as chaperone and possessing antiapoptotic activities (31–34). The Cryab is ubiquitously expressed in various layers of retina in mice and is abundantly expressed in RGCs; it has crucial roles in the survival of RGCs and other neuronal cells (46, 48, 49). In this investigation, we demonstrated that optical neuropathy phenotype was manifested by the CRYAB p.E105K mutation in heterozygosity in 17 participants (9 males and 8 females) of 42 members among 3 Han Chinese families with dominantly inherited form. These 17 affected participants bearing the p.E105K mutation revealed the unprecedented combination of dominant inheritance and LHON-like phenotype in these families (2, 9, 16, 17). However, other CRYAB mutations including p.R120G and p.S153F mutations are associated with a broad variety of neurological, cardiac, and muscular disorders, while some mutations including p.P20S mutation caused the sole clinical phenotype such as dominant cataracts (50–57). This discrepancy, that different mutations in the CRYAB gene caused different phenotypes, may reflect to the characteristics of mutations or some extent the different expression pattern of CRYAB gene. Indeed, the p.E105K mutation resided at a highly conserved ACD (residues 66–148) involved in the oligomerization of proteins and regulating the chaperone-like function (35, 36). The in vitro assays showed that the p.E105K mutation at ACD perturbed the secondary and tertiary structures of CRYAB, decreased stability, changed conformation, and reduced formation of oligomers, as in the case of p.R120G mutation in the ACD (52, 58, 59). Furthermore, the p.E105K mutation resulted in decreasing chaperone activity of CRYAB, evidenced by faster aggregation rate and earlier starting time of aggregation using a model aggregation assay (38, 50). The p.E105K mutation reduced the formation of oligomers and changed the conformation of CRYAB. This was strong evidence that p.E105K mutation–induced CRYAB structure and function alterations contributing to the pathogenesis of optical neuropathy.

The interaction of CRYAB with cytochrome *c* or VDAC and mitochondrial location of this protein indicated the effects of p.E105K mutation on both apoptotic process and mitochondrial function (34, 40, 53). Here, the immunoprecipitation assay revealed decreased interaction of CRYAB p.E105K with cytochrome *c* or VDAC. The p.E105K mutation promoted the apoptotic process, evidenced by increasing ratios of annexin V^+^ cells, elevated releases of cytochrome *c* into cytosol, and increased levels in caspases-9 in both MT cell lines bearing the p.E105K mutation and knockdown cell lines, as in the cases of cells bearing the LHON-associated mtDNA mutations (15, 30, 60–62). However, the levels in the BAX/BCL-XL remained unchanged in MT cell lines or knocked-down cell lines, as compared with control cell lines. These data suggest that CRYAB mutation affected the apoptosis by weaker interaction with cytochrome *c* but not with other apoptosis-activated or inhibited proteins BAX or BCL-XL.

The reduced interaction of p.E105K CRYAB with cytochrome *c* or VDAC may affect the stabilization of OXPHOS, with especially pronounced effects in the complex III. Reduced levels of CYTB and CYC1, subunits of complex III, were observed in the MT cell lines. The p.E105K mutation also caused the various decreases in other subunits of complex I, III, IV, and V, encoded by mtDNA or nuclear genes. These deficiencies gave rise to the instability of complexes I, III, IV, and V observed in MT cell lines. Each OXPHOS complex is a multisubunit machine integrated into the mitochondrial inner membrane, comprising mtDNA-encoded subunits and nuclear-encoded subunits, except complex II (63). These mtDNA-encoded subunits appear to act as seeds for building new complexes, which requires nucleus-encoded subunits to import and assemble with the assistance of assembly factors (63). In fact, a LHON susceptibility allele in the PRICKLE3 only affected the stability and assemble of complex V via the weaker interaction of MT PRICKLE3 and ATP8 (39). As a consequence, these defects resulted in the reduced activities of these respiratory chain enzyme complexes in the MT cell lines. The effect of CRYAB mutation on mitochondrial function was further supported by the fact that the CRYAB-knockdown cell line exhibited defective stability, assembly of OXPHOS, and reductions in basal OCR; ATP-linked OCR; proton leak OCR; maximal OCR; and reserve capacity. Furthermore, the CRYAB mutation affected mitochondrial dynamics, evidenced by abnormal mitochondrial morphology, increasing levels of fusion-related proteins OPA1 and MFN1, and reduced levels of fission-related proteins DRP1 and FIS1 in the in CRYAB-knockdown cells. These data highlight the effect of CRYAB mutation on mitochondrial function.

We investigated the biochemical and pathological consequences of CRYAB defects in retinas using the Cryab^E105K^-knockin mouse model, generated by the CRISPR/Cas9 gene-editing approach. Both Cryab^+/105K^ and Cryab^105K/105K^ mice were viable and developed the retinal deficiency at 8 weeks old. Cryab^+/105K^ and Cryab^105K/105K^ mice exhibited the decreases in RGCs in retina ganglion layers, as in the case of the loss of RGCs in the OPA1, Nduf4, and Prickle3 MT mice (39, 64–66). The RGC degeneration was further supported by axonal swelling and degeneration in the optic nerves of both Cryab^+/105K^ and Cryab^105K/105K^ eyes, similar to those carrying LHON-linked ND6^P25L^ mutation (67). Furthermore, the Cryab^+/105K^ and Cryab^105K/105K^ mice exhibited abnormal retinal vasculatures that were tortuous and dilated, with thinner vessels, comparable with those in mouse models of dominant optical neuropathy (65, 66). Finally, the Cryab^+/105K^ and Cryab^105K/105K^ mice displayed the retinal dysfunction, including the reductions in the scotopic b-wave of dark-adapted amplitude and photopic b-wave ERG amplitude for cone function of Cryab MT mouse eyes. However, the Cryab^+/105K^ and Cryab^105K/105K^ mice did not reveal the phenotype of hereditary myopathy and cataract observed in Cryab-R120G-knockin mice model (45). Characteristics of mutations in Cryab may account for different disorders. The effect of these mutations may depend on its location within the protein and lead to different functional consequences, thus representing a molecular mechanism for pleiotropy (68). Here, Cryab-knockin mice recapitulated the biochemical phenotypes in cell lines derived from patients bearing the CRYAB p.E105K mutation. In particular, Cryab^E105K^ mice displayed the pronounced decreases of Cryab protein and increased levels of cytochrome *c* and apoptosis in Cryab^E105K^ mice. Furthermore, Cryab^E105K^ mice exhibited the aberrant stability, assemble, and activity of OXPHOS, especially in pronounced effects in the complex III. These mitochondrial defects were further supported by abnormal mitochondrial morphology, observed in the MT retina (Supplemental Figure 7). Therefore, we concluded that the Cryab-knockin mouse recapitulated the clinical phenotypes in patients carrying the CRYAB mutation.

In summary, we identified a potentially novel molecular component of retinal ganglion cells, and its dysfunctions led to defects in ganglion neurons and consequently caused optic atrophy. Here, we demonstrated that the CRYAB mutation led to optic atrophy through pleiotropic effects including mitochondrial dysfunction, defective chaperone-like function, and impaired apoptosis. The CRYAB-knockin mouse exhibited the mitochondrial dysfunction and impaired apoptosis, consistent with those in patients bearing the CRYAB mutation. CRYAB-knockin mouse recaptured clinical phenotypes with the retina deficiencies. Our findings provide insights into pathophysiology of hereditary optic neuropathy arising from defects in major lens proteins and provide a step toward therapeutic interventions for this disorder.

## Methods

### Sex as a biological variable.

Lymphocytes from both males and female lineage members were included in our study. Animals examined in our study were distributed into different experimental groups by genotypes, with a similar ratio of males and females. No obvious differences were observed by sex.

### Families, individuals, and cell lines.

DNA samples used for this investigation were from 17 vision-impaired and 25 non–vision-impaired members of 3 Han Chinese families, 761 genetically unrelated probands with optical neuropathy, and 316 unrelated Chinese non–vision-impaired individuals (13, 14, 29). The ophthalmic examinations and other clinical evaluations of probands, other members of these families, and control individuals were conducted as detailed elsewhere (13, 14, 29).

Lymphoblastoid cell lines generated from members of the WZ1303 pedigree (vision-impaired members III-7 [male, 26] and III-8 [male, 34] carrying the heterozygous c.313G>A mutation; non–vision-impaired individuals III-5 [female, 31] and III-9 [male, 32]) were grown in RPMI 1640 medium (Invitrogen, Thermo Fisher Scientific), supplemented with 10% FBS. The SH-SY5Y cell line and dermal fibroblast cell lines were grown in DMEM (Corning Inc.), supplemented with 10% FBS.

### Sequencing and genetic data analysis.

Whole exome sequencings of 4 members (III-7, II-2, II-1, and II-8) of WZ1303 pedigree were performed by BGI. The data for these whole exome sequences were submitted into BioProject database (ID: PRJNA634625). High-quality genomic DNA (3 μg) was captured by hybridization using the SureSelect XT Human All Exon 50 Mb kit (Agilent Technologies). Samples were prepared according to the manufacturer’s instructions. Each captured library was run on a HiSeq 2000 instrument, and sequences were generated as 90 bp pair-end reads. An average of 82 million paired reads was generated per sample, the mean duplication rate was 13.45%, and 98.64% of targeted regions were covered by at least 70× mean depth. All sequencing reads were mapped to the human reference genome (GRCh37) at University of California, Santa Cruz (Santa Cruz, California, USA). Software SOAPsnp was used to assemble the consensus sequence and call genotypes in the target regions. GATK (IndelGenotyper V1.0) was used for indel detection. The threshold for filtering single-nucleotide polymorphisms (SNPs) included the following criterion: SNP quality score should be ≥ 20, sequencing depth should be between 4 and 200, estimated copy numbers should be no more than 2, and the distance between 2 SNPs should be larger than 5. SNPs from these analyses were summarized in Supplemental Table 3. Variants were annotated by ANNOVAR. To further filter the SNP, the criteria for potential candidate variants were nonsynonymous or in splice sites within 6 bp of an exon, with less than 1% MT allele frequency in variant databases, and cosegregated with the phenotype. The mutations were validated by Sanger sequencing in all family members and other unrelated samples. Primers of CRYAB for Sanger sequencing are listed in Supplemental Table 4, including the primers for the genotyping of the c.313G>A mutation in exon 2. The entire mtDNA of Chinese individuals was analyzed as described elsewhere (69). The resultant sequences were compared with the CRYAB genomic sequence (RefSeq NC_000011.9; https://www.ncbi.nlm.nih.gov/nuccore/NC_000011.9/) and the updated consensus Cambridge sequence (GenBank accession no. NC_012920) (70).

### Western blot assays.

Western blot assays were performed using 20 μg of total cellular proteins isolated from human cell lines or mouse tissues, as detailed elsewhere (71). These antibodies used for this investigation are summarized in Supplemental Table 5. Peroxidase AffiniPure goat anti–mouse IgG and goat anti–rabbit IgG (Beyotime, A0216 and A0208, respectively) were used as secondary antibodies, and protein signals were detected using the ECL system (CWBIO). The quantification of density in each band was performed as detailed elsewhere (72).

### Construction of recombinant pEGFP-N1-CRYAB plasmid.

The full-length human CRYAB cDNAs were amplified with primers — 5′- CCGCTCGAGATGGACATCGCCAT -3′ (forward) and 5′- CGACCGGTGGGTATTTCTTGGGG -3′ (reverse) — and cloned into the XhoI and AgeI restriction sites of pEGFP-N1 vector (Addgene plasmid 172281).

### Immunofluorescence analysis.

Cells were cultured on glass coverslips (0.1 mg/mL poly-d-lysine coated for 2 hours before seeding suspension-cultured cells), fixed in 4% formaldehyde for 15 minutes, permeabilized with 0.2% Triton X-100, blocked with 5% FBS for 1 hour, and immunostained with specific primary antibodies overnight at 4°C. Subsequently, cells were washed with phosphate-buffered saline (PBS) and then incubated for 1 hour with either Alexa Fluor 488 goat anti–mouse IgG (H+L; Abcam, ab150113) or Alexa Fluor 594 goat anti-rabbit IgG (H+L; Abcam, ab150080). Cells were then washed again in PBS, counterstained with DAPI solution, and mounted with Fluoromount (Sigma-Aldrich). Cells were examined using a confocal fluorescence microscope (Olympus Fluoview FV1000).

### Molecular dynamics simulations.

The ACD of CRYAB dimer (PDB ID: 2klr) was chosen as the initial WT model in our simulations. The coordinates of p.E105K mutation at the ACD of dimer were generated by Pymol. Simulations were conducted with Gromacs 4.5.5 using CHARMM36 force field parameters. The proteins were solvated in a cubic box that contains 32 Na^+^ and 32 Cl^–^ to maintain a concentration of 50 mM NaCl, and ions were added to neutralize each system. Energy minimization employing the Steepest Descent algorithm was carried out for 4 rounds to make contact favorable, and the system energy was minimized until the maximum force was lower than 100 kJ/mol/nm except for the final rounds which set to 10 kJ/mol/nm. Then 4 rounds of equilibration were performed with positional constraints, which were applied on h-bonds of heavy atoms, main-chain atoms, Cα atoms, and no atoms using LINCS algorithm successively. The canonical ensemble (NVT, a statistical ensemble that is used to study material properties under the conditions of a constant particle number N, constant volume V, and temperature T fluctuating around an equilibrium value) of 100 ps from 50 K to 300 K was used in the first step, while the constant-pressure and constant-temperature ensemble (NPT, a statistical ensemble that is used to study material properties under the conditions of a constant particle number N, a pressure P fluctuating around an equilibrium value, and a temperature T fluctuating around an equilibrium value) was performed with a time step of 2 femtoseconds (fs) to equilibrate the solvent and whole system on the rest steps. The temperature and pressure of NPT ensemble were maintained at 300,000 and 1 bar by the v-rescale method and Berendsen barostat. The electrostatic interactions were calculated by the Particle-Mesh Ewald algorithm. MD simulations of 500 ns followed the equilibrated system were produced with a time step of 2 fs. Gromacs 4.5.5 packages were used to analyze ACD dimer and their MT trajectories. RMSD and root mean square fluctuation (RMSF) fluctuating curves were obtained from R version 3.4.1. The trajectory of each system was observed with molecular visualization program VMD, and the figures related to structures of ACD dimer were generated by Pymol.

### Cloning and purification of CRYAB.

The full-length coding region of CRYAB cDNA was obtained by reverse transcription PCR amplification using the high-fidelity Pfu DNA polymerase (Promega) and total RNA isolated from lymphoblastoid cell lines derived from the proband III-7 and married-in control III-5 as a template, with primers with NcoI site, 5′- CATGCCATGGACATCGCCATCCAC -3′, and XhoI site, CCGCTCGAGTTTCTTGGGGGCTGC -3′ (nt.1152-1177) (GenBank accession no. NM_139169.5). The PCR products were cloned by using the TA Cloning Kit (TAKARA), analyzed by Sanger sequencing, and then subcloned into a pET28α vector (Addgene plasmid 46970). Recombinant WT and MT CRYAB-His fusion proteins were expressed in *E. coli* BL21 (DE3) cells. Cultures of transformed cells were induced with 400 μM IPTG when the optical density at 600 nm reached 0.6 and incubated at 16°C for 8 hours. After incubation, cells were harvested by centrifugation at 4,000*g* for 20 minutes and suspended in 1/25 culture volume of lysis buffer (50 mM NaH_2_PO_4_, 300 mM NaCl, 10 mM imidazole). The suspension was sonicated on ice, and insoluble material was removed by at 8,000*g* for 20 minutes at 4°C. The protein purification was performed following the Ni-NTA Superflow Cartridge Handbook (QIAGEN).

### Immunoprecipitation assay.

The full-length human CRYAB cDNAs were amplified with primers — 5′-CGGAATTCATGGACATCGCCATCC-3′ (forward) and 5′- CCGCTCGAGTTTCTTGGGGGCTGC-3′ (reverse) — and cloned into the EcoRI and XhoI restriction sites of pcDNA3.1-HA vector or pcDNA3.1-CRYAB-FLAG vector. HEK293T cells (2.0 × 10^6^) were transfected with 12 μg of pcDNA3.1-CRYAB-HA or pcDNA3.1-CRYAB-FLAG for 36 hours using Hieff Trans liposomal transfection reagent following the manufacturer’s protocol (Yeasen). The cells were harvested and suspended with 1 mL of lysis buffer (1% NP40; 50 mM Tris–HCl [pH 7.6]; 150 mM NaCl; 1× Protease Inhibitor Cocktail [Bimake]) and lysed on ice for 30 minutes. The lysates were centrifuged at 20,000*g* for 10 minutes at 4°C. The supernatants were incubated with 10 μL of beads (cross-linked to 25 μg of monoclonal antibody, anti-HA [Abclonal, AE008], anti-FLAG [Abclonal, AE005], anti–cytochrome *c* [Proteintech, 10993-1-AP], anti-CRYAB [Cell Signaling Technology, 45844S], or VDAC [Proteintech, 55259-1-AP]) overnight at 4°C with rotation. Beads were washed 4 times and were then boiled for 5 minutes after SDS loading was added. Finally, the IP fractions were analyzed by Western blot analysis.

### Generation of CRYAB-knockdown cell lines.

The dermal fibroblast derived from non–vision-impaired participant SD IV-4 lacking the mtDNA and CRYAB mutations were cultured in DMEM, supplemented with 10% FBS. The shRNA oligo primers targeting CRYAB were: forward, 5′- CCGGTGTGATTGAGGTGCATGGAAACTCGAGTTTCCATGCACCTCAATCACATTTTTG -3′; reverse, 5′- AATTCAAAAATGTGATTGAGGTGCATGGAAACTCGAGTTTCCATGCACCTCAATCACA-3′. Pairs of shRNA were cloned into PLKO.1 TRC (Addgene). pLKO.1 vectors with shRNA or scramble shRNA were cotransfected into dermal fibroblasts with psPAX2 and pMD2.G for lentivirus production. Complete medium was changed after 4–6 hours of transfection. The virus was collected 48 hours after transfection and centrifuged at 12,000*g*. Cells were cultured in the medium containing virus for 48 hours. After selection with puromycin at 1 μg/mL concentration for another 24 hours, the cells were subjected to the Western blot analysis to examine the levels of CRYAB before using for various assays.

### Annexin V/PI apoptosis assay by flow cytometry.

For discrimination of apoptotic and nonapoptotic cells by annexin V/PI staining, cells were harvested and stained with annexin V and 1 μL of propidium iodide (PI) (V13242, Thermo Fisher Scientific) according to the manufacturer’s instruction. Each sample was detected by NovoCyte (ACEA Biosciences) and analyzed using NovoExpress software (72).

### Mitochondrial function assays.

BN-PAGE and in-gel activity assay were performed by using mitochondrial proteins isolated from various cell lines, as detailed elsewhere (41, 42). The enzymatic activities of respiratory chain complexes I, II, III, and IV from various cell lines were assayed as described previously (42). The OCRs of various cell lines were measured via extracellular flux analyzer (43).

### Knockin mice.

C57BL/6J mice were originally purchased from Shanghai SLAC Laboratory Animal Co Ltd. Sanger sequencing analysis of the Crb1 gene failed to detect the Rd8 mutation in vendor lines of C57BL/6J mice (73). The generation of CRYAB p.E105K-knockin mice was performed by CRISPR/Cas9 system, as detailed previously (74–76). The oligo 5′ GTCCACGGCAAGCACGAAGAACGCCAGGTGTG-3′ was chosen as targeting guide RNA. mRNA of in vitro transcribed Cas9 and chimeric sgRNA as well as a 99 bp single-stranded oligodeoxynucleotide were injected into zygotes of C57BL/6 mouse. After injection, surviving zygotes were transferred into the oviducts of pseudo pregnant females. Obtained F0 mice were validated by sequencing using primer pairs: Cryab-F: 5′- CTCCTCTCCTCCTCCTGTCC -3′, Cryab-R: 5′- CGAGCCCTAGCTTAGCTTCC -3′. Mice with the expected single-nucleotide mutation were crossed with WT (Cryab^+/+^) C57BL/6 mice to produce F1-knockin mice. Offspring bearing p.E105K mutation were labeled as Cryab^+/105K^ and Cryab^105K/105K^, respectively. Sequence of the ssOND for knockin mouse generation was 5′- AGTCAAGGTTCTGGGGGACGTGATTGAGGTCCACGGCAAGCACAAAGAACGCCAAGTGTGTGGACCTCTCCGTCCTCTTTTGTGAATCCACTTTGTGCA -3′.

### Optical coherence tomography.

Optical coherence tomography was carried out as detailed elsewhere (77). Briefly, mice were anesthetized by i.p. injection of 2.5% pentobarbital at a dose of 10 μL per 1 g mouse body weight, and the pupils were dilated with the compound tropicamide. Ofloxacin Eye Ointment was applied to the corneal surface. Optical Coherence Tomography images were taken using an image-guided tomographer (Micron IV-OCT2; Phoenix Research Laboratories); OCT circle scan was measured using the Reveal OCT2 system, and the image was captured centered on the optic nerve head (ONH).

### Fluorescein angiography.

Fluorescence angiography of eyes in Cryab^+/+^, Cryab^+/105K^, and Cryab^105K/105K^ mice (at 8 weeks) was carried out using the Micron III camera (Phoenix Research Laboratories Inc.), as described elsewhere (77). Pupils were dilated with 1% tropicamide (Bausch & Lomb) followed by the application of GenTeal Lubricant Eye Gel (Alcon). Systane lubricant eye drops (Alcon) were applied to keep the cornea moist. Mouse pupils were then i.p. injected with 0.2% AK-FLUOR (Akorn) at a dose of 0.1 mL per 10 g of mouse body weight. Photos were taken with a camera containing a barrier filter for fluorescein angiography.

### IHC.

Mouse eyes at the age of 8 weeks were fixed for 24 hours in FAA (formaldehyde [37%]/ethanol [95%]/acetic acid in a 1:4:0.5 ratio), and sectioned in the midsagittal plane at 3 μM. The sections were placed on glass slides and were deparaffinized and hydrated with xylene and graded alcohol. The sections were preincubated in a boiled sodium citrate buffer for antigen retrieval and were immunostained with specific primary antibodies overnight at 4°C. Subsequently, sections were washed with PBS and then incubated for 1 hour with HRP-conjugated anti–rabbit IgG (Beyotime, A0208), visualized with 3,3′-diaminobenzidine to produce brown staining, and counterstained with hematoxylin. Images were taken by Leica DM4000B-M.

Axonal ultrastructural analysis. Mouse eyes at the age of 8 weeks were fixed in 2.5% glutaraldehyde for 24 hours and postfixed with 2% osmium tetroxide for 2 hours. The samples were dehydrated with increasing concentrations of ethanol (50%, 70%, 90%, and 100%) and transferred to absolute acetone for 20 minutes. After placing in 1:1 mixture of absolute acetone and the final Spurr resin (SPI-CHEM, 02690-AB) mixture for 1 hour, the samples were transferred to 1:3 mixture of absolute acetone and the final resin mixture for 3 hours and to final Spurr resin mixture overnight. Sections (1 μm) were stained with 1% paraphenylenediamine for 10 minutes and washed by ethanol. Five points in each optic nerve were photographed with Leica Microsystems CMS GmbH microscope using a 100× objective lens. One picture in each set was excluded based on highest degree of longitudinally arranged axonal fibers. The nerves in the 4 remaining pictures were manually counted (67).

### Statistics.

Statistical analysis was performed using GraphPad Prism (version 8.0.2). Statistical analyses for evaluating differences between 2 groups were performed using 2-tailed paired and unpaired Student’s *t* test. For evaluating significance in more than 2 groups, 1-way ANOVA followed by Bonferroni’s post hoc test were used. *P* values of less than 0.05 were considered to be statistically significant.

### Study approval.

Informed consent, blood samples, and clinical evaluations were obtained from all participants and families, under protocols approved by the Ethic Committees of Zhejiang University School of Medicine. All animal care and study protocols used in this investigation were approved by Zhejiang University IACUC.

### Data availability.

Values for all data points in graphs are available in the supplemental Supporting Data Values file. Representative experiments are shown in the figures and supplemental materials.

## Author contributions

MXG designed the experiments, monitored the project progression, and performed data analysis and interpretation. Chenghui Wang, LZ, and ZN performed the biochemical analyses. Chenghui Wang, ML, HL, CA, and JZ performed the whole exome sequence and mutational screening. Chenghui Wang, ZN, and Chunyan Wang carried out the mouse experiments. ML, JZ, and YJ carried out the clinical evaluation and recruited patients with LHON. QY and YG performed molecular dynamics simulations. HL and YJ analyzed the genetic data. Chenghui Wang prepared the initial draft of the manuscript. MXG made the final version of the manuscript. All authors reviewed the manuscript.

## Supplementary Material

Supplemental data

Unedited blot and gel images

Supporting data values

## Version 1 11/19/2024

In-Press Preview

## Version 2 01/09/2025

Electronic publication
