# Supplementary material for: Mutation of CRYAB encoding a conserved mitochondrial chaperone and antiapoptotic protein causes hereditary optic atrophy
Source: JCI Insight. 2024 Nov 19;10(1):e182209. doi: 10.1172/jci.insight.182209 (PMC11721302; doi:10.1172/jci.insight.182209)
Supplement: Supplemental data [file jciinsight-10-182209-s280.pdf]

Supplementary data

Mutation of *CRYAB* encoding a conserved mitochondrial chaperone and anti-apoptotic protein causes hereditary optic atrophy

Chenghui Wang[1,2,3#], Liyao Zhang[2#], Zhipeng Nie[1,2,3#], Min Liang[4], Hanqing Liu[2], Qiuzi Yi[2], Chunyan Wang[2], Cheng Ai[1,2,3], Juanjuan Zhang[4], Yinglong Gao[2,5], Yanchun Ji[2,5], and Min-Xin Guan[1,2,3,6]*

The supplemental data included the following information:

1. Supplemental Figure 1, 2, 3, 4, 5, 6, and 7
2. Supplemental Table 1, 2, 3, 4 and 5

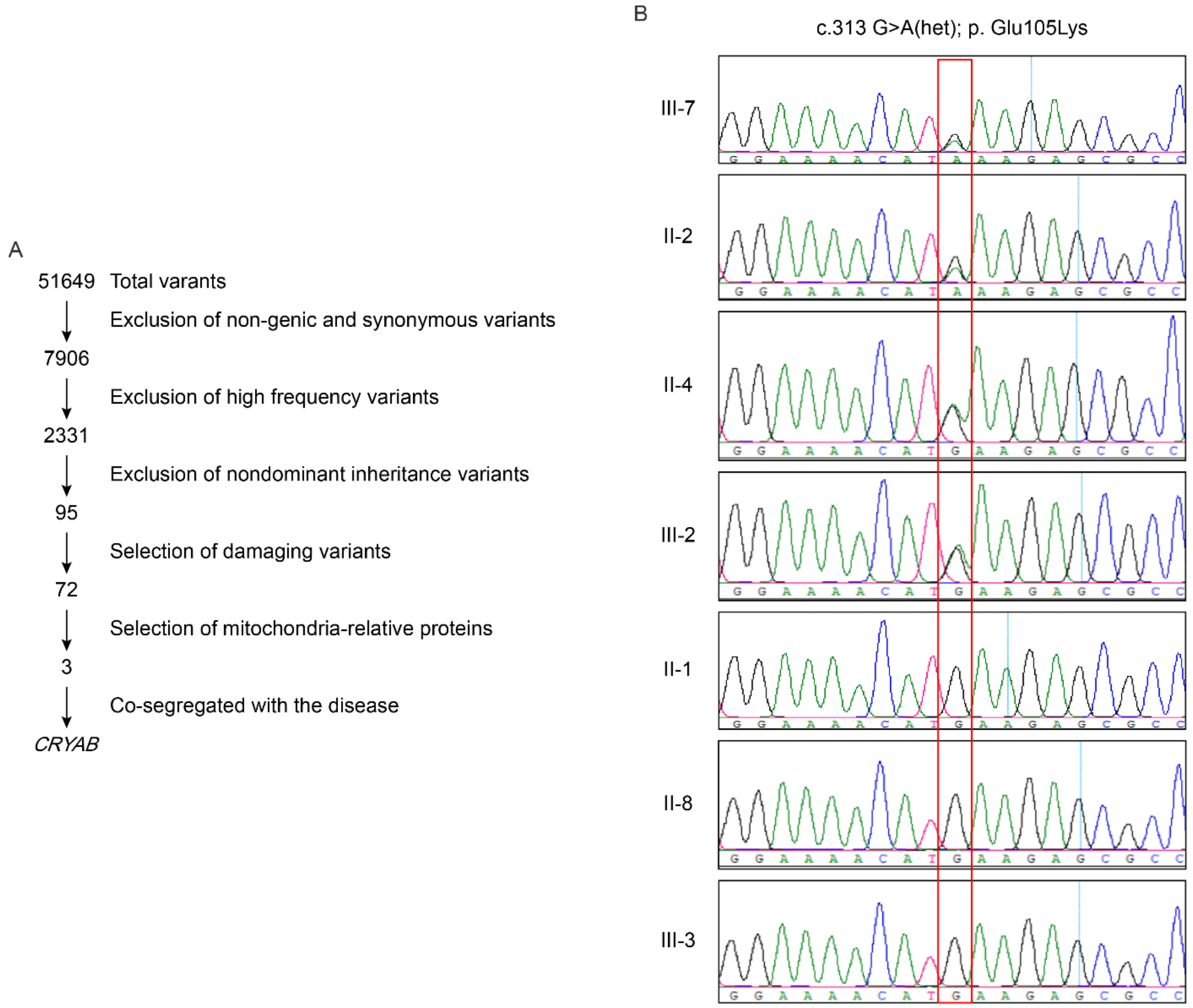

Supplemental Figure 1 (related to Figure 1). Identification of c.313G>A (p.Glu105Lys) mutation in *CRYAB* gene. Summary of whole exome sequencing of the proband (WZ1303-III-7). The identified single nucleotide variant (SNV) c.313G>A (p.Glu105Lys) is located in *CRYAB*, a gene encoding a major lens protein belonging to the small heat-shock family of proteins and possessing anti-apoptotic activities. (**B**) Partial sequence chromatograms of *CRYAB* gene. Sanger sequencing of affected individuals III-7, II-2, II-4, III-2 and unaffected individuals II-1, II-8, III-3 of the WZ1303 family. The red frame indicates the location of the nucleotide changes at position 313.

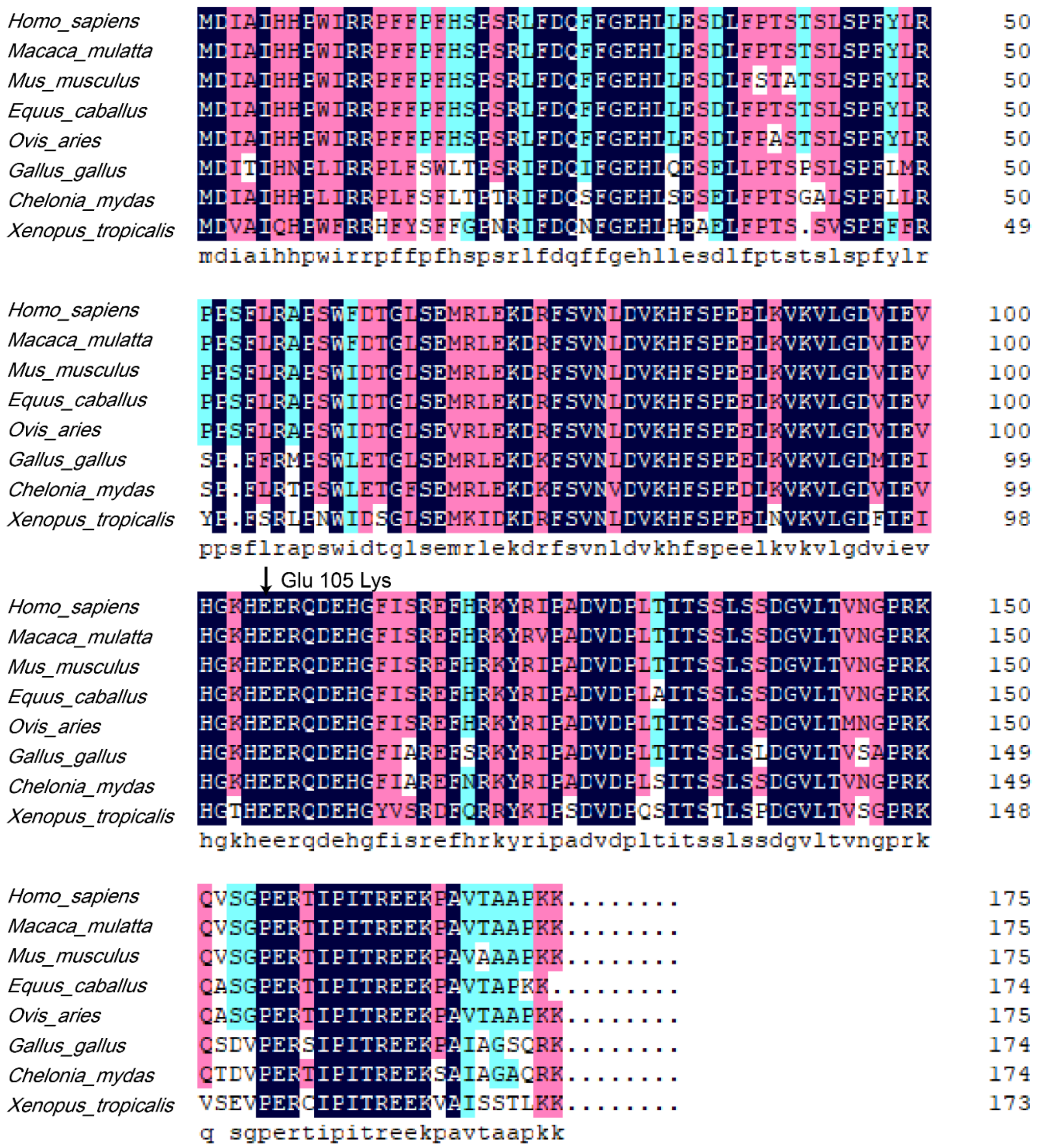

Supplemental Figure 2 (related to Figure 1). Alignments of the amino acid sequences of CRYAB family among different species. The alignment was generated using the DNAMAN software. The organisms and corresponding accession numbers used for this analysis are as follows: *Homo sapiens* (NP_001276737.1), *Macaca mulatta* (XP_028688504.1), *Mus musculus* (CAJ18549.1), *Equus caballus* (XP_001501829.1), *Ovis aries* (NP_001012475.1), *Gallus gallus* (NP_990507.2), *Chelonia mydas* (XP_007072715.3) and *Xenopus tropicalis* (XP_002932964.1). Numbers give the position of residues in proteins in relation to the first methionine of the Homo sapiens. Amino acid residues shaded black are identical; those shaded pink and faint blue are similar in at least six residues and four residues of eight homologs, respectively.

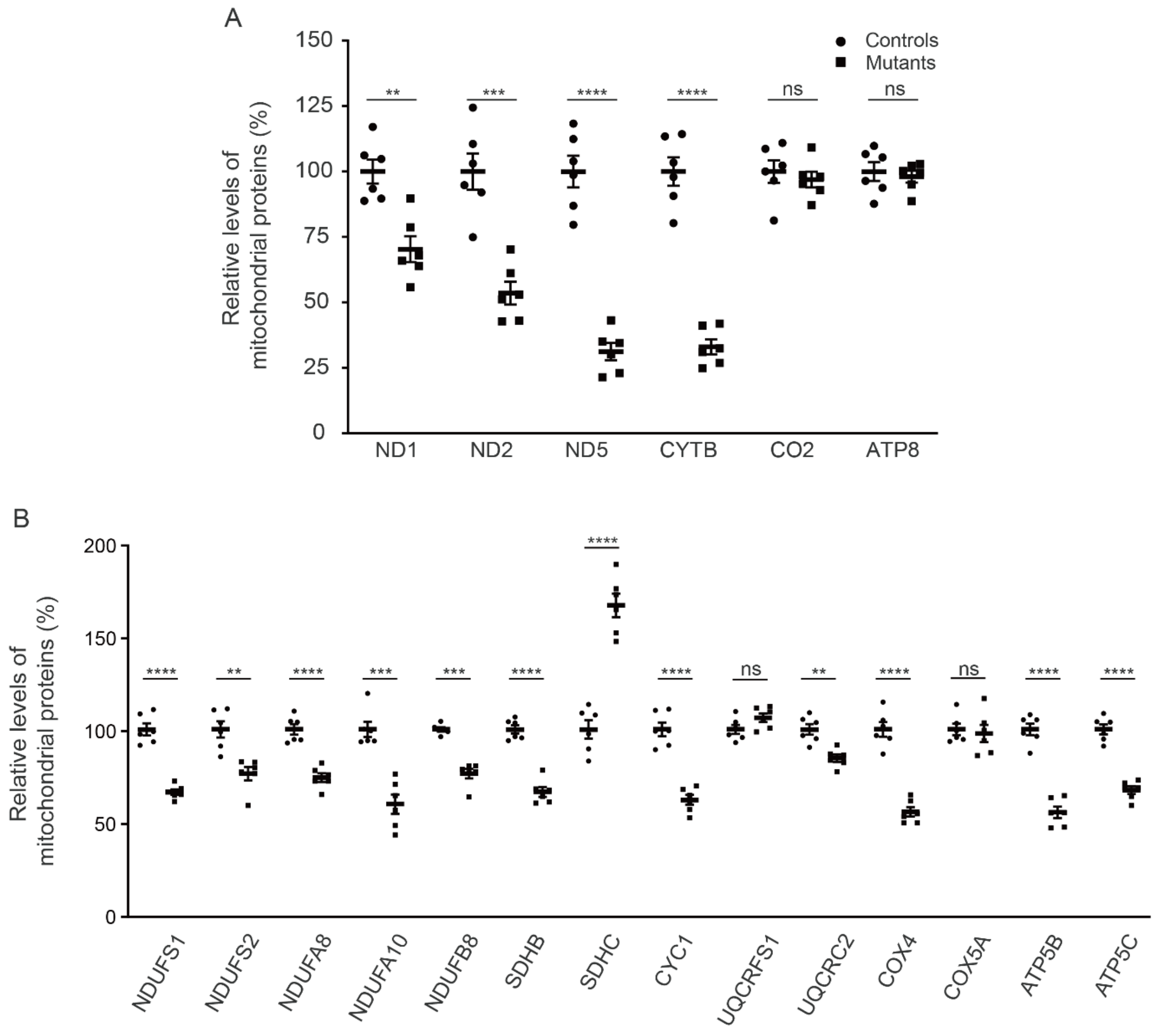

Supplemental Figure 3 (related to Figure 4): Quantification of OXPHOS subunits. (A) Quantification of mtDNA encoding subunits: ND1, ND2, ND5, CYTB, CO2 and ATP8 in mutant and control cell lines. **(B)** Quantification of nucleus-encoding subunits: NDUFS1, NDUFS2, NDUFA8, NDUFA10, NDUFB8, SDHB, SDHC, CYC1, UQCRFS1, UQCRC2, COX4, COX5A, ATP5B and ATP5C in mutant and control cell lines. Data are as shown as mean ± SEM of triplicates. *P* indicates the significance, according to the t-test, of the differences between mutant and control cell lines. *$P < 0.05$; **$P < 0.01$; ***$P < 0.001$; ****$P < 0.0001$; ns, not significant.

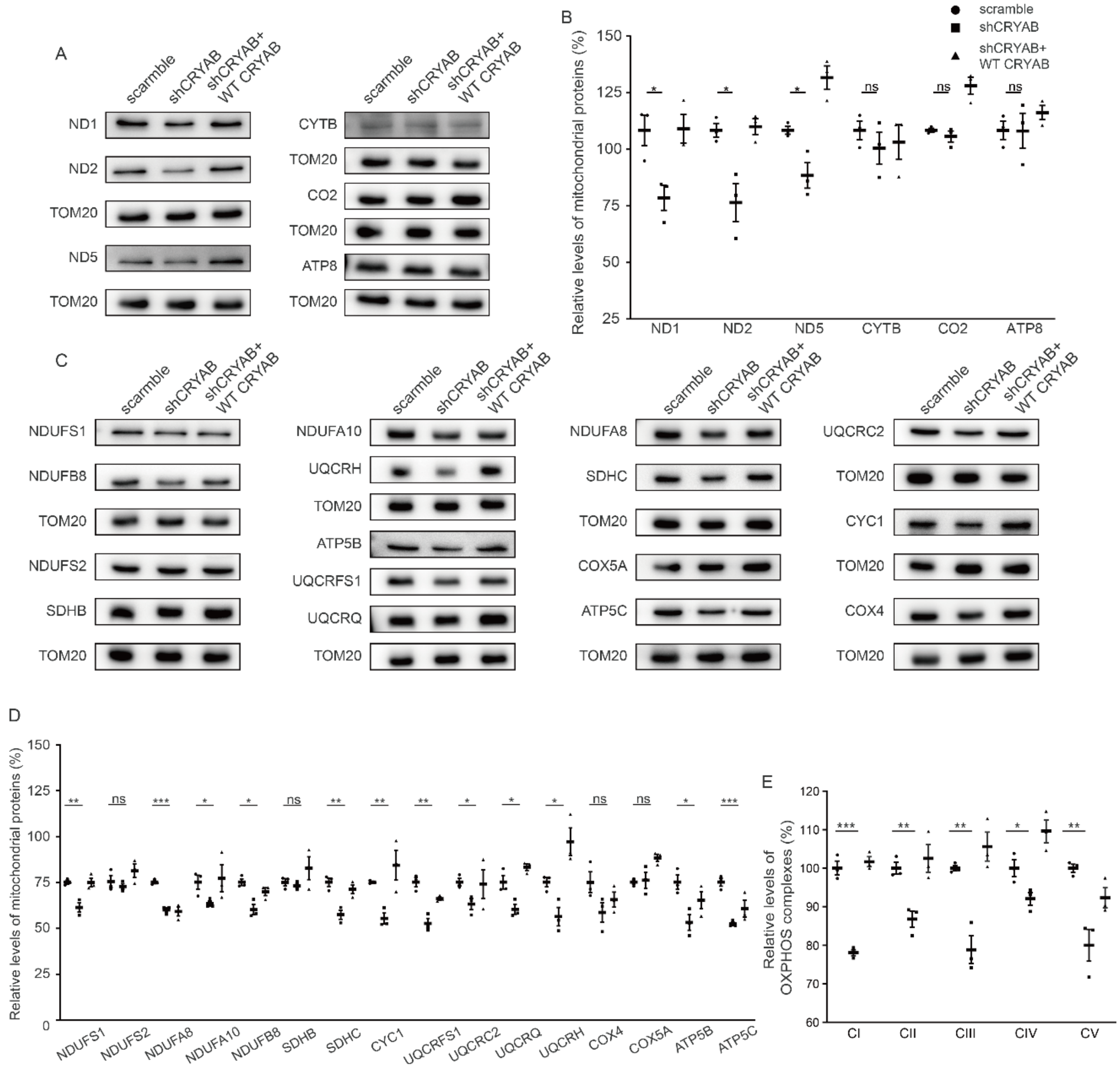

Supplemental Figure 4 (related to Figure 4): Western blotting analysis of OXPHOS subunits from *CRYAB* knock-down cell line. (A and C) Twenty micrograms of total cellular proteins from various cell lines were electrophoresed through a denaturing polyacrylamide gel, electroblotted, and hybridized with antibodies for 22 subunits of OXPHOS (6 encoded by mtDNA and 16 encoded by nuclear genes), and TOM20 as a loading control, respectively. (B and D) Quantification of mitochondrial proteins: 6 mtDNA-encoding subunits (B) and 16 nucleus-encoding subunits (D). Average relative each polypeptide content per cell was normalized to the average content per cell of TOM20 in each cell line. The values for the latter are expressed as percentages of the average values for the WT cell line. (E) Average levels of subunits from each complex of OXPHOS (8 of complexes I, 2 of II, 6 of III, 3 of IV, and 3 of V). The calculations were based on three independent determinations. Data are as shown as mean ± SEM of triplicates. $^{*}P < 0.05$; $^{**}P < 0.01$; $^{***}P < 0.001$; $^{****}P < 0.0001$; ns, not significant.

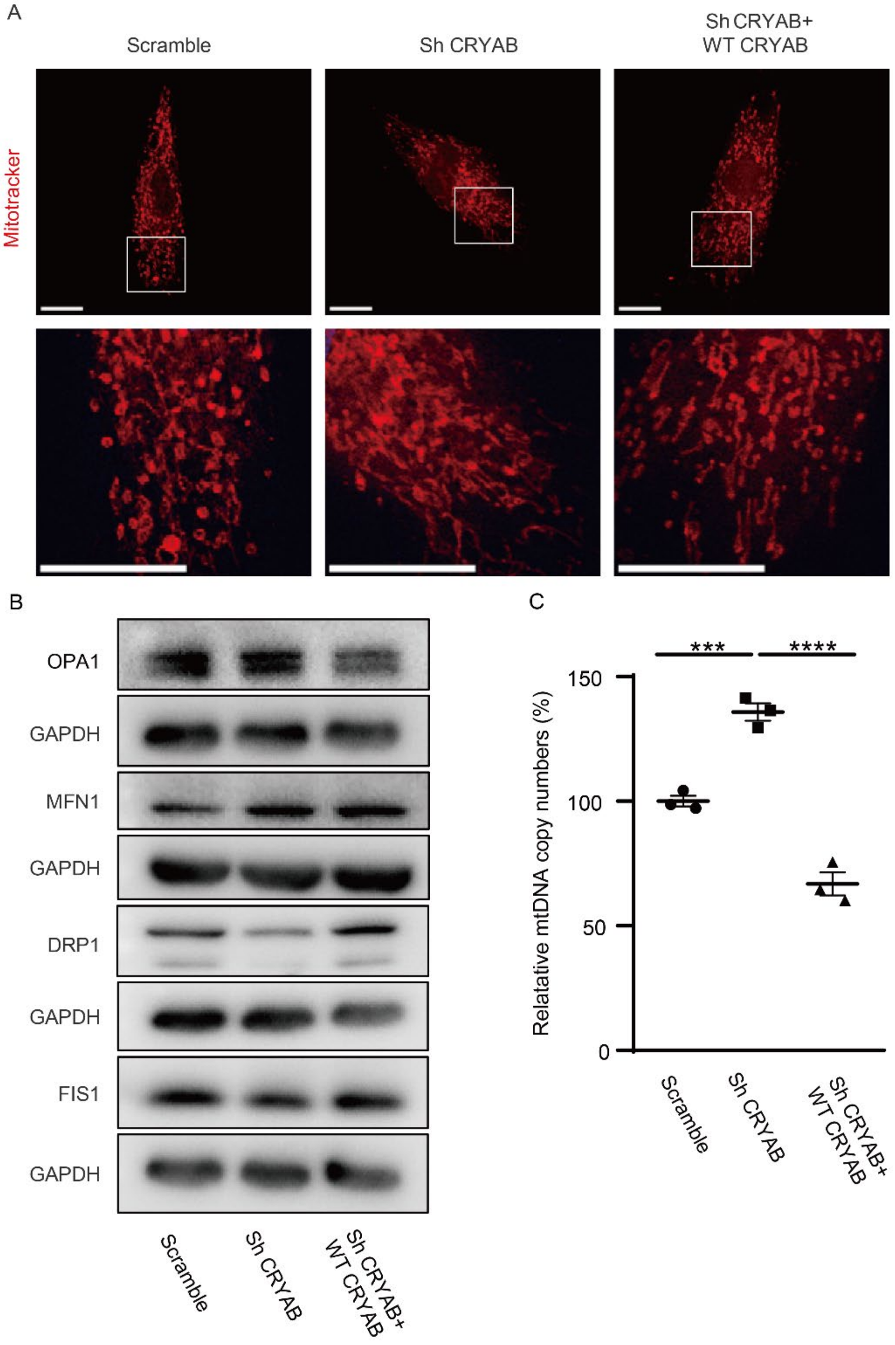

Supplemental Figure 5 (related to Figure 4): Assessment of mitochondrial dynamics and mtDNA contents of CRYAB knock-down cell lines. (A) Mitochondria from CRYAB knock-down cell lines were visualized by immunofluorescent staining with MitoTracker Red. Scale bar = 20um. (B) Western blot analysis of mitochondrial fusion-associated proteins (MFN1, OPA1) and mitochondrial fission-associated proteins (DRP1, FIS1) among CRYAB knock-down cell lines, with GAPDH as a loading control. (C) Measurement of mtDNA contents by qPCR. Mitochondrial DNAs from mutant and control cell lines were normalized to β-actin encoded by nuclear gene (71). The calculations were based on three independent experiments. Data are as shown as mean ± SEM of triplicates. *P* indicates the significance, **P*<0.05, ***P*<0.01, ****P*<0.001; ns, no statistically significant by one-way ANOVA followed by Bonferroni's post hoc test.

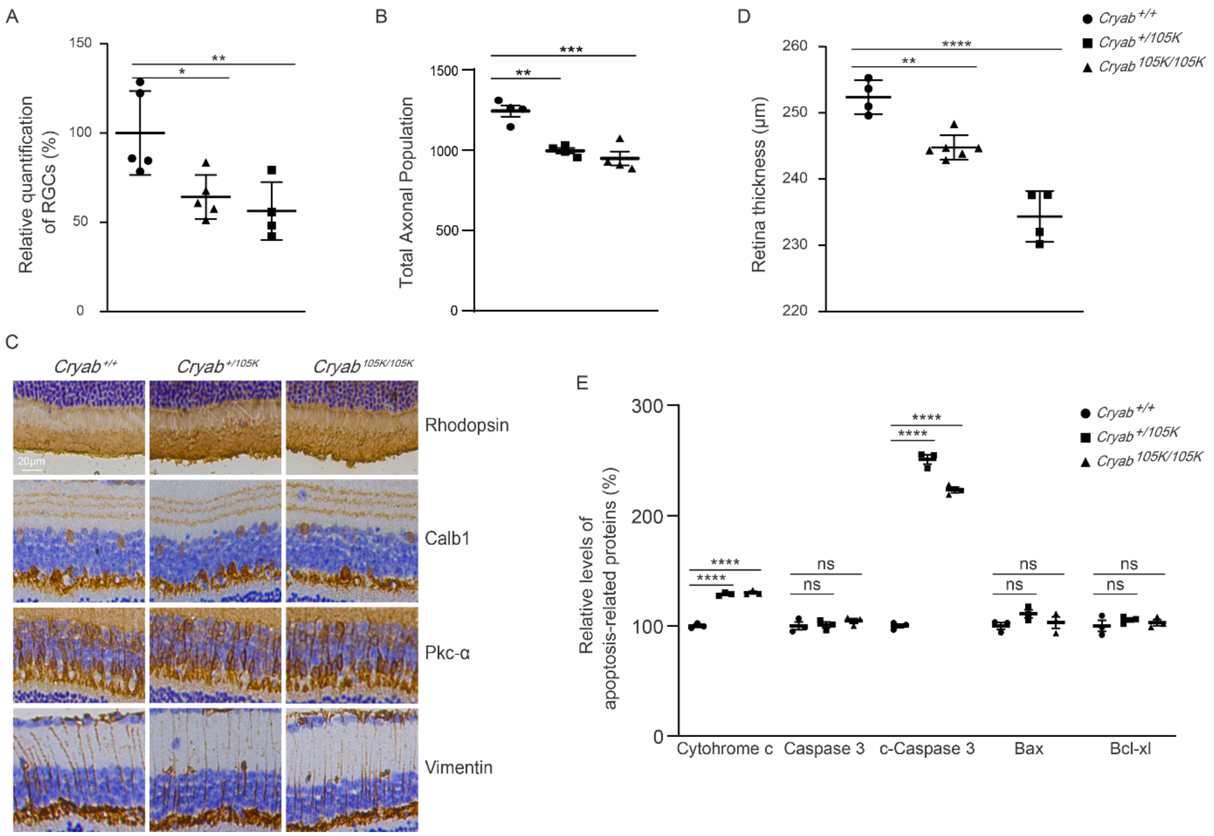

Supplemental Figure 6 (related to Figure 5 and 6): (A) Relative quantification of retinal ganglion cells. (B) Axonal counts from mice optic nerve cross-section of Cryab$^{+/+}$, Cryab$^{+/105K}$ and Cryab$^{105K/105K}$. Five points in each nerve were photographed using a 100× objective lens. One picture in each set was excluded based on highest degree of longitudinally arranged axonal fibers. Four remaining pictures were manually counted. N=4. **(C)** Immunohistochemistry of mouse retina. Rhodopsin^{+} for rod photoreceptor, Calb1^{+} for horizontal cells, Pkc-α^{+} for bipolar cells, and Vimentin^{+} for Müller cells. **(D)** Quantification of mouse retinal thickness. **(E)** Quantification of apoptosis-related proteins. The calculations were based on three independent determinations. Data are as shown as mean ± SEM of triplicates. *P* indicates the significance, **P*<0.05, ***P*<0.01, ****P*<0.001; ns, no statistically significant by one-way ANOVA followed by Bonferroni's post hoc test.

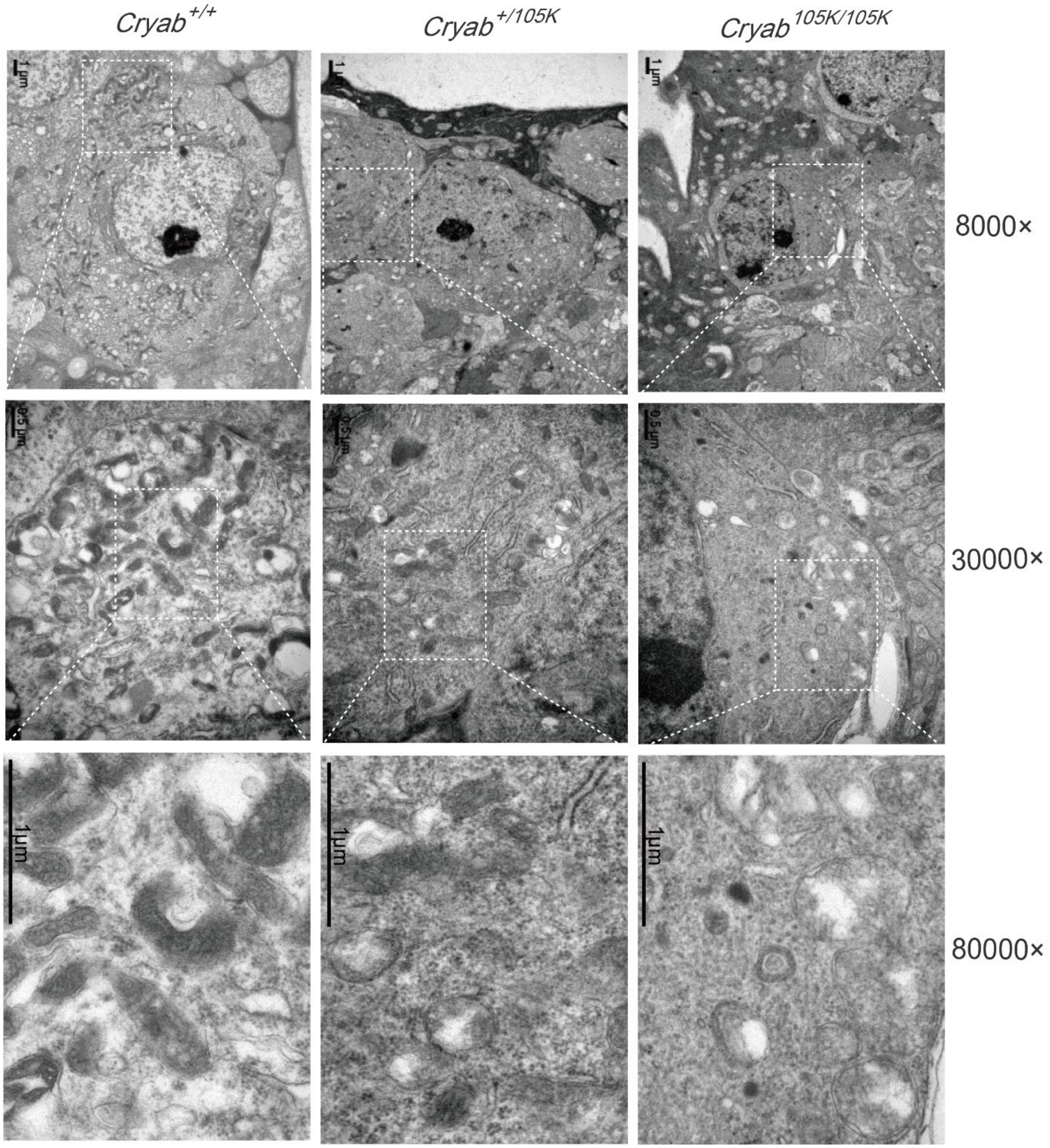

Supplemental Figure 7 (related to Figure 5 and 6): Mitochondrial morphologies in RGCs from WT and MT mice at 8 weeks of age by transmission electron microscopy. Ultrathin sections were visualized with 8000×, 30000×, and 80000× magnifications. The white rectangular dashed frame is the enlarged area. Scale bars: 1 μm.

Supplemental Table 1: mtDNA variants in 3 Han Chinese probands with optic atrophy

Gene	Position	Replacement	AA change	Conservation (H/B/M/X)#	CRS‡	WZ1303 III-7	TZ008 III-1	TZ206 III-1	Previously Reported†
D-loop	73	A-G			A	G	G	G	Yes
	150	C-T			C	T			Yes
	210	A-G			A		G		Yes
	249	del A			A			del	Yes
	263	A-G			A	G	G	G	Yes
	310	T-TC			T	TC	TC	TC	Yes
	489	T-C			T	C			Yes
	16093	T-C			T			C	Yes
	16129	G-A			G			A	Yes
	16140	T-C			T		C		Yes
	16164	A-G			A	G		G	Yes
	16172	T-C			T	C		C	Yes
	16182	A-C			A	C			Yes
	16183	A-C			A	C	C		Yes
	16189	T-C			T	C	C		Yes
	16304	T-C			T			C	Yes
	16399	A-G			A			G	Yes
	16519	T-C			T		C	C	Yes
12S rRNA	709	G-A		G/A/A/-	G		A		Yes
	750	A-G		A/A/A/-	A	G	G	G	Yes
	752	C-T		C/C/A/-	C	T			Yes
	1107	T-C		T/C/T/T	T	C			Yes
	1438	A-G		A/A/A/G	A		G	G	Yes
16S rRNA	2706	A-G		A/G/A/A	A	G	G	G	Yes
	3537	A-G	Syn		A		G		Yes
MT-ND1	3528	C-T	Syn		C	T			Yes
	3970	C-T	Syn		C			T	Yes
	4086	C-T	Syn		C			T	Yes
MT-ND2	4769	A-G	Syn		A	G	G	G	Yes
	4883	C-T	Syn		C	T			Yes
	5178	C-A	Leu-Met	L/T/T/T	C	A			Yes
	5301	A-G	Ile-Val	I/L/M/L	A	G			Yes
	6392	T-C	Syn		T			C	Yes
	6960	C-T	Syn		C		T		Yes
MT-COX1	6962	G-A	Syn		G			A	Yes
	7028	C-T	Syn		C	T			Yes
	8291-9	del					del		Yes
MT-ATP6	8584	G-A	Ala-Thr	A/V/V/I	G		A		Yes
	8701	A-G	Thr-Ala	T/S/L/Q	A	G			Yes
	8860	A-G	Thr-Ala	T/A/A/T	A	G	G	G	Yes
	9053	G-A	Ser-Asp	S/G/G/T	G			A	Yes
	9180	A-G	Syn		A	G			Yes
MT-COX3	9540	T-C	Syn		T	C			Yes
	9548	G-A	Syn		G			A	Yes
	9950	T-C	Syn		T		C		Yes
	10310	G-A	Syn		G			A	Yes
	10325	G-A	Syn		G		A		Yes
MT-ND3	10397	A-G	Syn		A	G			Yes
	10398	A-G	Thr-Ala	T/T/T/A	A	G	G		Yes
	10400	C-T			C	T			Yes
MT-ND4	10873	T-C	Syn		T	C			Yes
	11380	A-G	Syn		A			G	Yes
	11719	G-A	Syn		G	A	A	A	Yes
	11944	T-C	Syn		T	C			Yes
	12026	A-G	Ile-Val	I/I/M/L	A	G			Yes
MT-ND5	12705	C-T	Syn		C	T			Yes
	12882	C-T	Syn		C			T	Yes
	13759	G-A	Ala-Thr	A/T/T/I	G			A	Yes
	13928	G-C	Ser-Thr	S/T/S/T	G		C		Yes
MT-CYB	14766	C-T	Thr-Ile	T/S/T/S	C	T	T	T	Yes
	14783	T-C	Syn		T	C			Yes
	15043	G-A	Syn		G	A			Yes
	15235	A-G	Syn		A		G		Yes
	15301	G-A	Syn		G	A			Yes
	15326	A-G	Thr-Ala	T/M/I/I	A	G	G	G	Yes

# Conservation among 4 species: *Homo sapiens* (H), *Bos taurus* (B), *Mus musculus* (M) *and Xenopus laevis* (X);
‡ CRS: Cambridge reference sequence;
† See online mitochondrial genome database: http://www.mitomap.org.

Supplemental Table 2 (related to Figure 1). Summary of clinical data for members of 3 Han Chinese families with optic atrophy

Subject	Gender	Age of test (year)	Age of onset (year)	Vision acuity (Right/Left eyes)	Level of vision impairment	*CRYAB* c.313G>A mutation
WZ1303-I-1	M	82	/	1.1/1.2	normal	+/+
WZ1303-I-2	F	80	21	0.05/0.08	moderate	+/-
WZ1303-II-1	M	62	/	1.2/1	normal	+/+
WZ1303-II-2	F	61	24	0.1/0.1	moderate	+/-
WZ1303-II-3	M	58	/	1.1/1.3	normal	+/+
WZ1303-II-4	F	57	20	0.1/0.2	mild	+/-
WZ1303-II-5	M	54	22	0.08/0.08	moderate	+/-
WZ1303-II-6	M	49	18	0.1/0.2	mild	+/-
WZ1303-II-7	F	48	/	1.0/1.1	normal	+/+
WZ1303-II-8	M	47	/	1.0/1.2	normal	+/+
WZ1303-II-9	F	45	/	1.3/1.1	normal	+/+
WZ1303-III-1	M	39	/	1.0/1.1	normal	+/+
WZ1303-III-2	F	38	14	0.1/0.08	moderate	+/-
WZ1303-III-3	F	35	/	1.2/1.0	normal	+/+
WZ1303-III-4	M	33	/	1.0/1.3	normal	+/+
WZ1303-III-5	F	31	/	1.0/1.0	normal	+/+
WZ1303-III-6	F	28	21	0.1/0.2	mild	+/-
WZ1303-III-7	M	26	17	0.1/0.1	moderate	+/-
WZ1303-III-8	M	34	19	0.2/0.2	mild	+/-
WZ1303-III-9	M	32	/	1.1/1.0	normal	+/+
WZ1303-III-10	M	27	/	1.1/1.1	normal	+/+
WZ1303-III-11	F	24	/	1.0/1.2	normal	+/+
WZ1303-III-12	M	20	15	0.1/0.2	mild	+/-
WZ1303-III-13	M	24	/	1.3/1.3	normal	+/+
WZ1303-III-14	F	22	/	1.0/1.0	normal	+/+
WZ1303-III-15	M	21	/	1.1/1.0	normal	+/+
WZ1303-IV-1	M	26	14	0.2/0.1	mild	+/-
WZ1303-IV-2	M	24	/	1.2/1.1	normal	+/+
WZ1303-IV-3	M	22	/	1.1/1.0	normal	+/+
WZ1303-IV-4	M	13	/	1.0/1.3	normal	+/+
TZ008- I-1	M	68	20	0.1/0.2	mild	+/-
TZ008- I-2	F	67	/	1.0/1.0	normal	+/+
TZ008-II-1	M	47	19	0.2/0.1	mild	+/-
TZ008-II-2	F	45	/	1.1/1.0	normal	+/+
TZ008-II-3	F	45	/	1.0/1.2	normal	+/+
TZ008-II-4	M	43	/	1.1/1.2	normal	+/+
TZ008-III-1	F	22	21	0.2/0.1	mild	+/-
TZ206- I-1	M	65	19	0.1/0.2	mild	+/-
TZ206- I-2	F	63	/	1.0/1.1	normal	+/+
TZ206- II-1	M	46	/	1.1/1.1	normal	+/+
TZ206- II-2	F	45	20	0.1/0.2	mild	+/-
TZ206-III-1	M	20	18	0.2/0.2	mild	+/-

F= female; M= male; The degree of visual impairment was defined according to the visual acuity as follows: normal > 0.3, mild=0.3-0.1; moderate = 0.1-0.05; severe = 0.05-0.02; and profound <0.02

Supplemental Table 3. Summary of exome sequencing data for four members of WZ1303 family

Categories	II-1	II-8	II-2	III-7
Number of genomic positions for calling SNPs	44078277	43686053	43948447	41839201
Number of high-confidence genotypes	40966044	40410162	41062675	38245214
Total number of SNPs	128413	107379	118940	110364
Synonymous –coding	12053	12107	12141	12027
Missense	11472	11653	11747	11591
Nonsense	108	118	109	103
Readthrough	12	10	8	12
Splice site	175	177	185	176
Intron	38510	36945	37822	37527
5' UTR	4086	3725	3775	3851
3' UTR	7888	6915	7495	7418
Intergenic	15898	15121	15984	15559
Homozygous	74710	61588	68904	63228
Heterozygous	53703	45791	50036	47136
Frame error	0	0	0	0

Supplemental Table 4. Oligonucleotides for Sanger sequence analysis of *CRYAB* gene

Primer names	Sequence (5'-3')	Description
CRYAB exon1-F	GAGCCACATAGAACGAAAG	Sequencing
CRYAB exon1-R	ATAAATGGGATACAGAGGACTA	Sequencing
CRYAB exon2-F	AAGCCCTACGAGGAAACA	Sequencing
CRYAB exon2-R	TGTTATGGCTTGGGACTG	Sequencing
CRYAB exon3-F	TCAGAACCTGTGCGTCAA	Sequencing
CRYAB exon3-R	TCCTGTTTATTGCCCTTG	Sequencing

Supplemental Table 5. Key Resources table

REAGENT or RESOURCE	SOURCE	IDENTIFIER
**Antibodies**		
CRYAB	Cell Signaling Technology	45844S
GAPDH	proteintech	10494-1-AP
DDDDK-Tag	abclonal	AE005
TOM20	abclonal	A19403
HA-Tag	abclonal	AE008
VDAC1/Porin	proteintech	55259-1-AP
Cytochrome *c*	proteintech	10993-1-AP
BAX	proteintech	50599-2-Ig
BCL-XL	proteintech	26967-1-AP
Caspase 9	proteintech	10380-1-AP
ND1	proteintech	19703-1-AP
ND2	proteintech	19704-1-AP
ND5	proteintech	55410-1-AP
CYTB	proteintech	55090-1-AP
CO2	proteintech	55070-1-AP
ATP8	proteintech	29398-1-AP
NDUFS1	proteintech	12444-1-AP
NDUFS2	abclonal	A12858
NSUFA8	abclonal	A12118
NDUFA10	abclonal	A10123
NDUFB8	proteintech	14794-1-AP
SDHB	proteintech	10620-1-AP
SDHC	abcam	Ab155999
CYC1	proteintech	10242-1-AP
UQCRFS1	proteintech	18443-1-AP
UQCRC2	proteintech	14742-1-AP
UQCRQ	proteintech	14975-1-AP
UQCRH	abclonal	A9395
COX4	proteintech	11242-1-AP
COX5A	proteintech	11448-1-AP
ATP5A	proteintech	14676-1-AP
ATP5B	proteintech	17247-1-AP
ATP5C	proteintech	10910-1-AP
GAPDH	GOOD HRER	AB-M-M001
Brn-3a	Santa Cruz	sc-8429
βIII-tubulin	Abcam	ab18207
Caspase 3	Proteintech	19677-1-AP
Cleaved-Caspase 3	Affinity	AF7022
OPA1	Abclonal	A9833
MFN1	Proteintech	13798-1-AP
DRP1	Proteintech	10242-1-AP
FIS1	Proteintech	12957-1-AP
**Software**		
Microsoft-Excle	Microsoft	
GraphPad Prism9	GraphPad Software	
**Oligonucleotides**		
Primers	This paper	See Table 4
