# Supplementary material for: Mutation of CRYAB encoding a conserved mitochondrial chaperone and antiapoptotic protein causes hereditary optic atrophy
Source: JCI Insight. 2024 Nov 19;10(1):e182209. doi: 10.1172/jci.insight.182209 (PMC11721302; doi:10.1172/jci.insight.182209)

Unedited gel images

Full unedited gel for Figure 1D

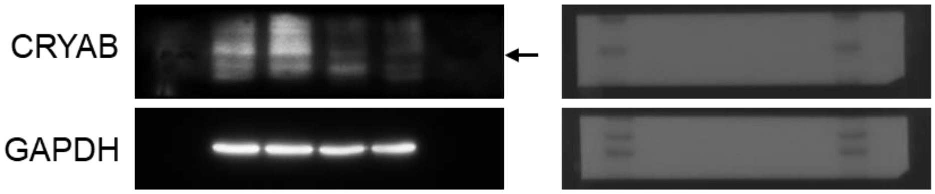

Full unedited gel for Figure 1D

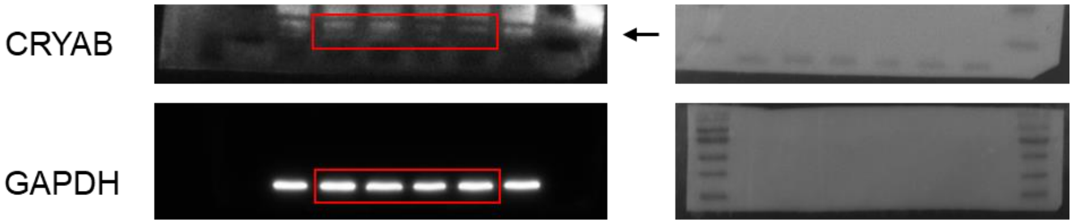

Full unedited gel for Figure 1E

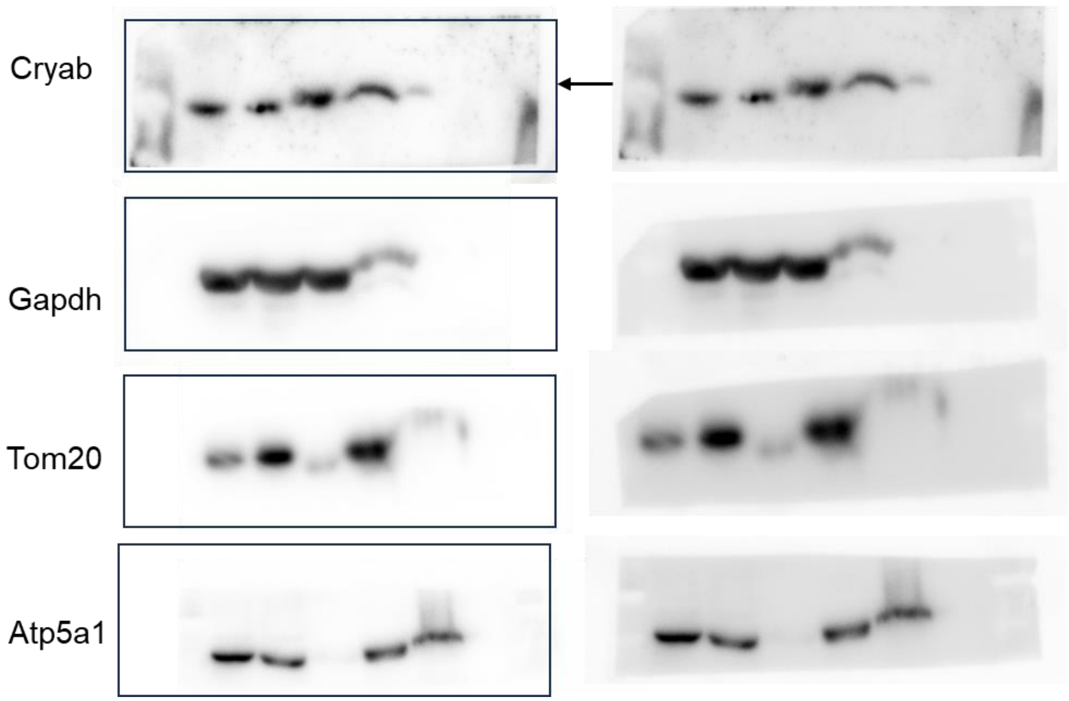

Full unedited gel for Figure 2C

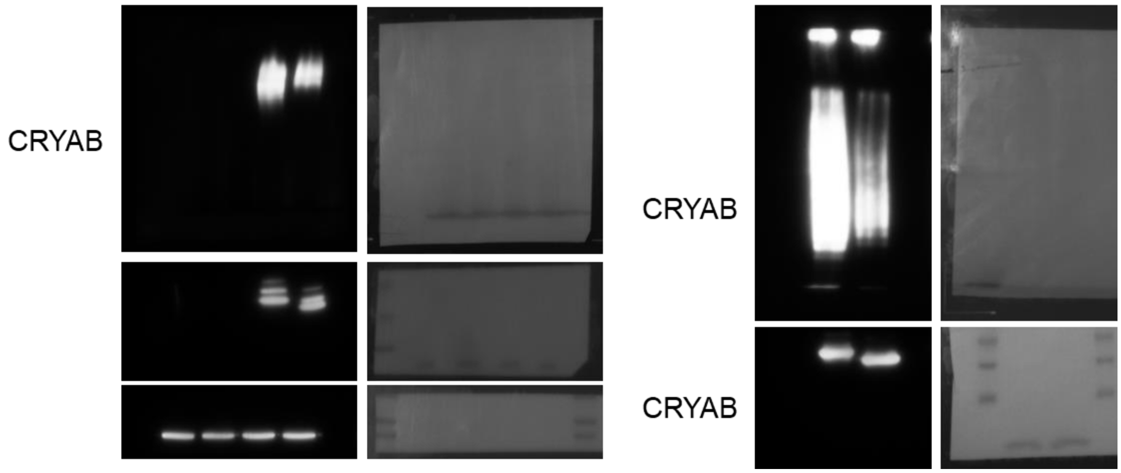

Full unedited gel for Figure 2E

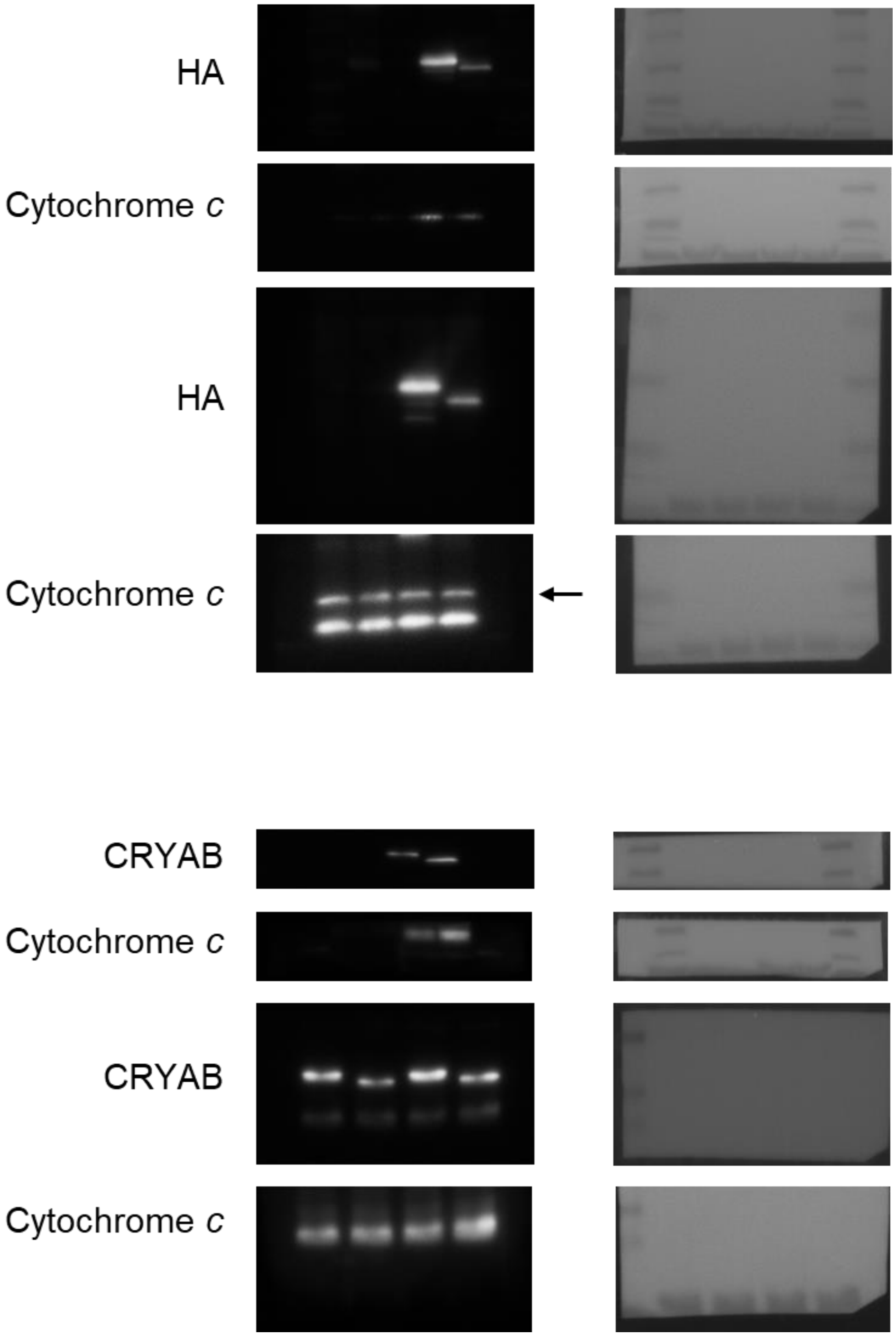

Full unedited gel for Figure 3C

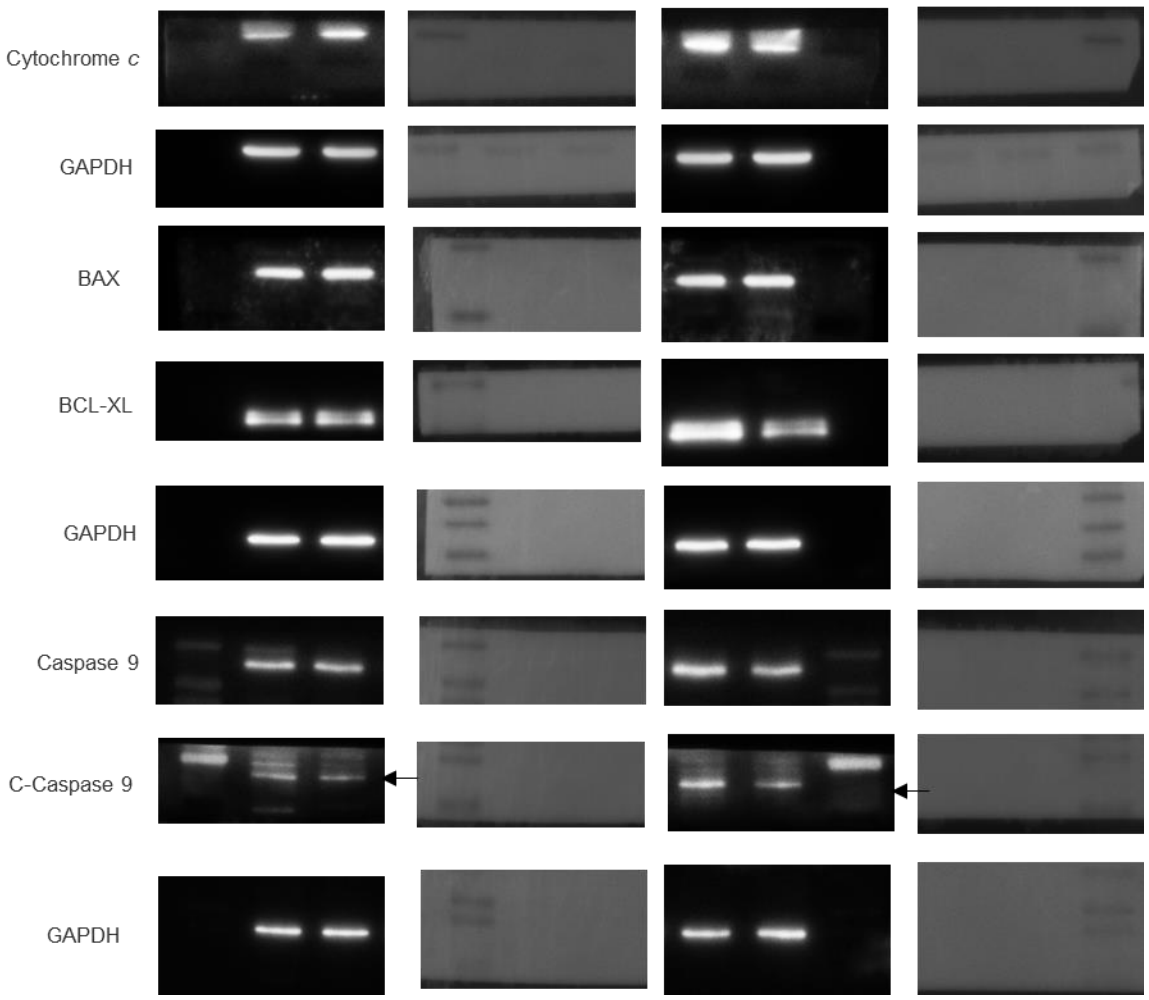

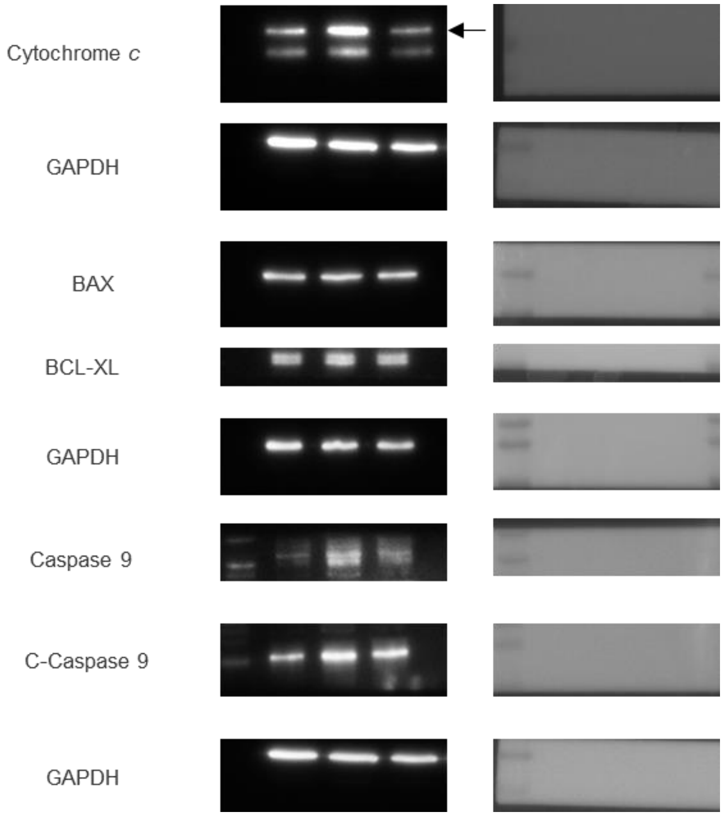
Cytochrome *c*
GAPDH
BAX
BCL-XL
GAPDH
Caspase 9
C-Caspase 9
GAPDH

Full unedited gel for Figure 4A

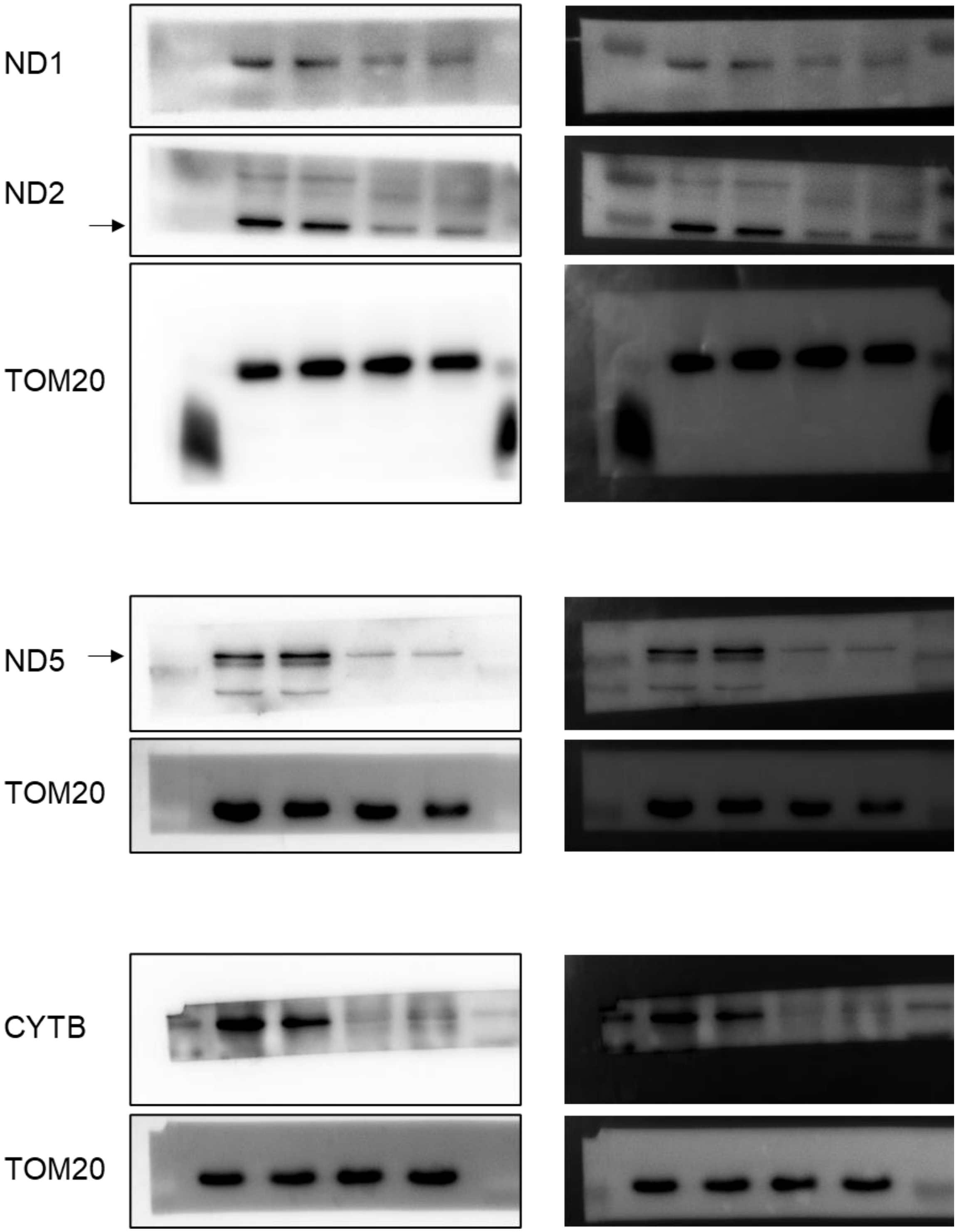

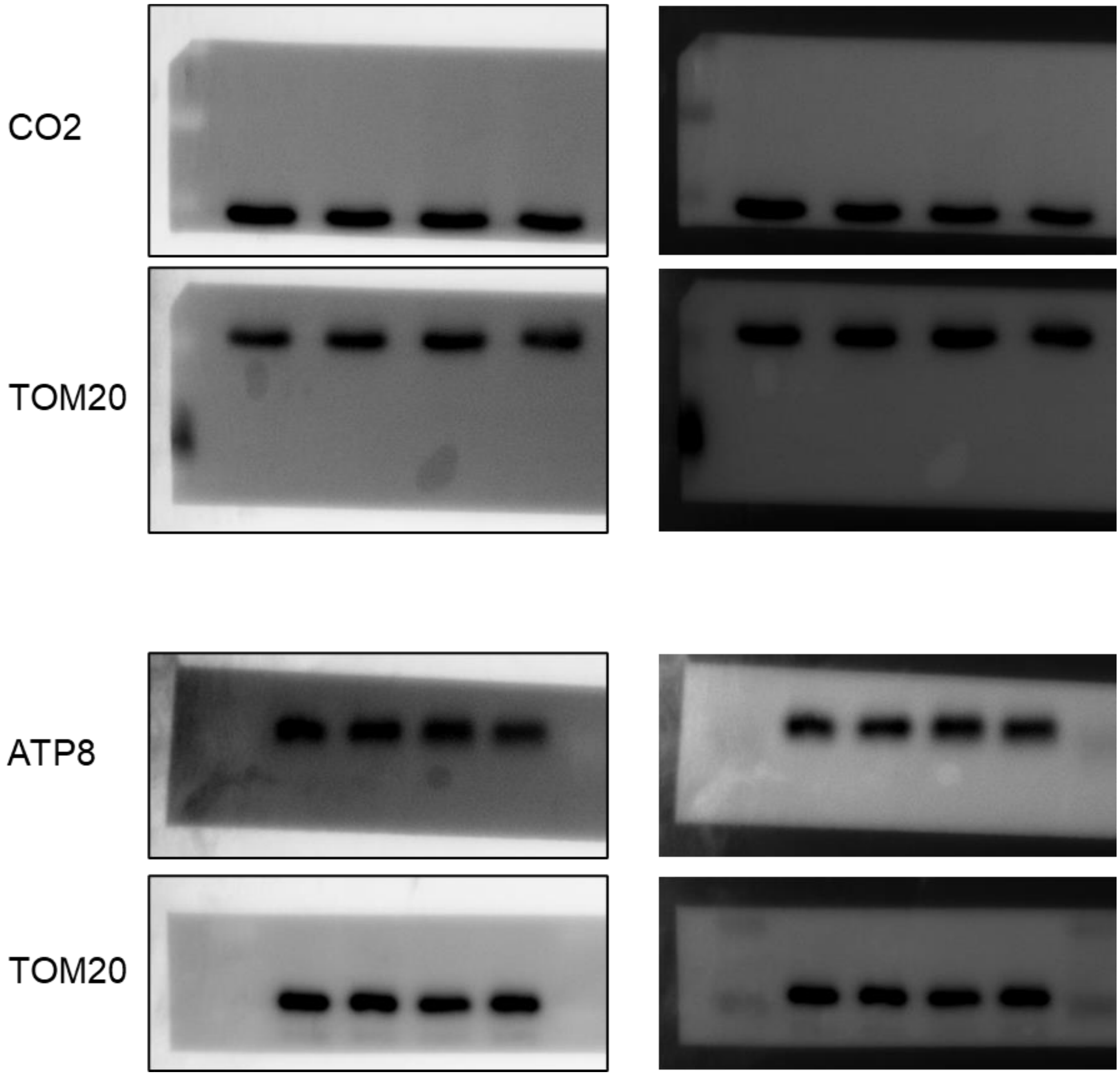
CO2
TOM20
ATP8
TOM20

Full unedited gel for Figure 4B

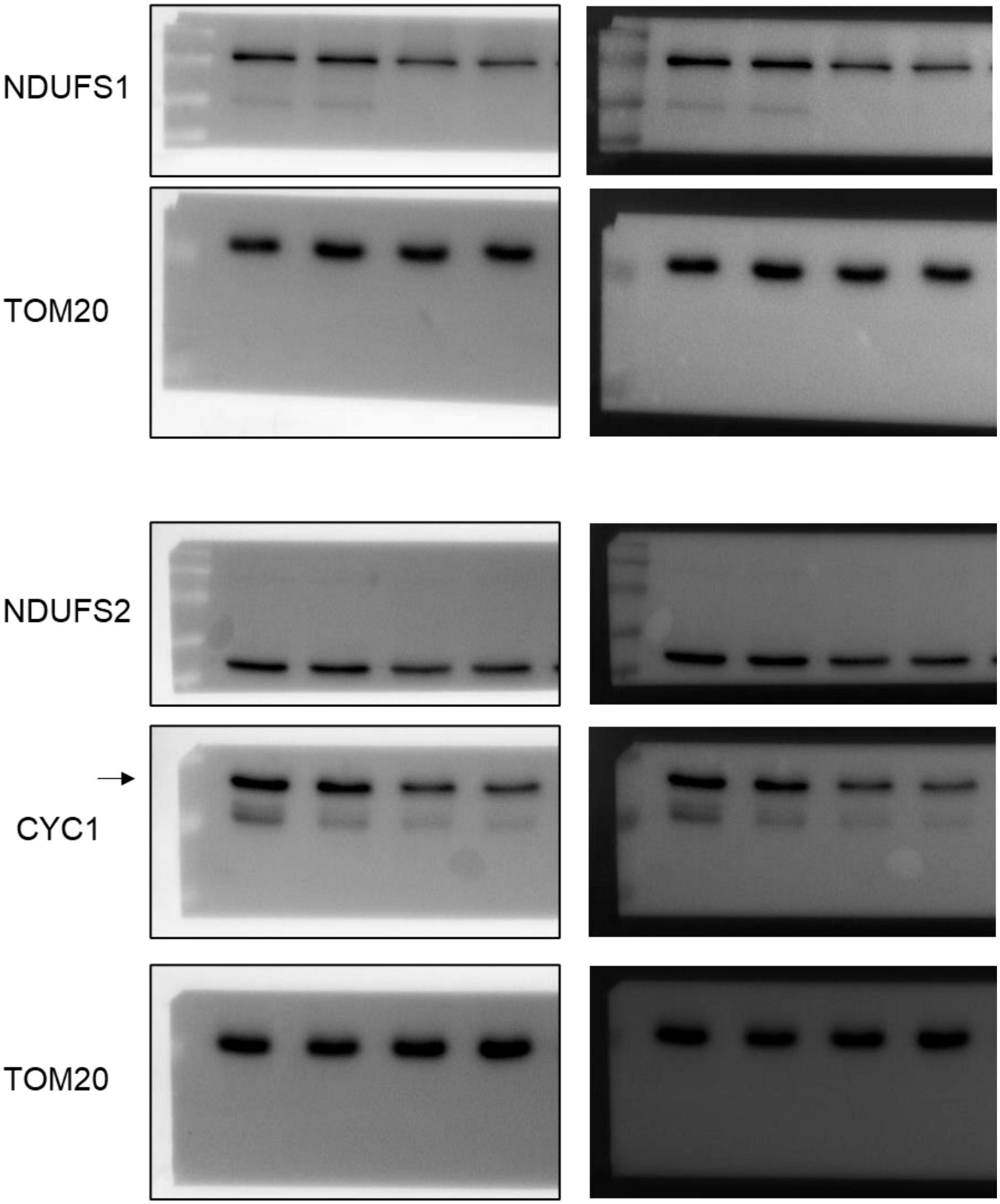

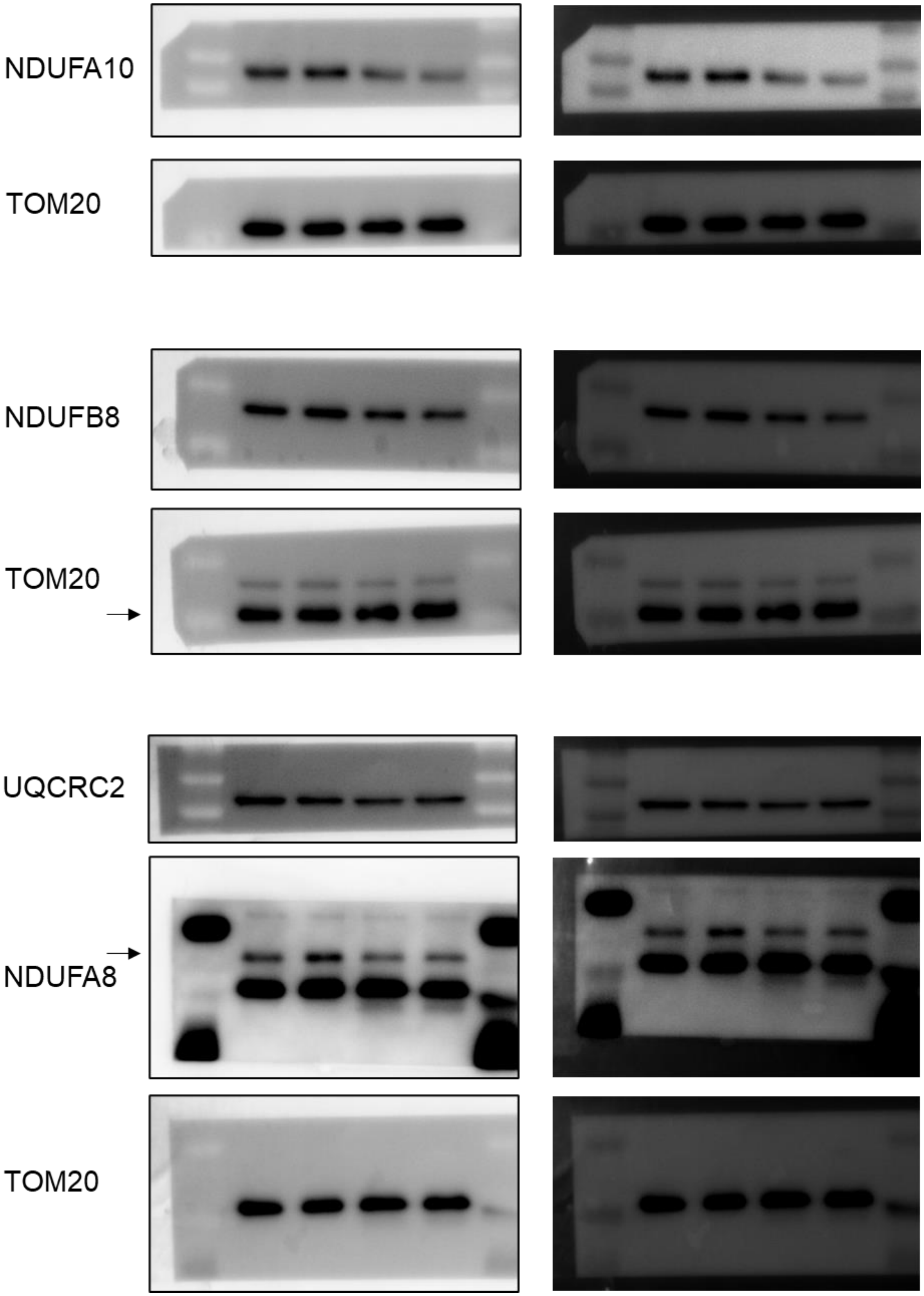
NDUFA10
TOM20
NDUFB8
TOM20
UQCRC2
NDUFA8
TOM20

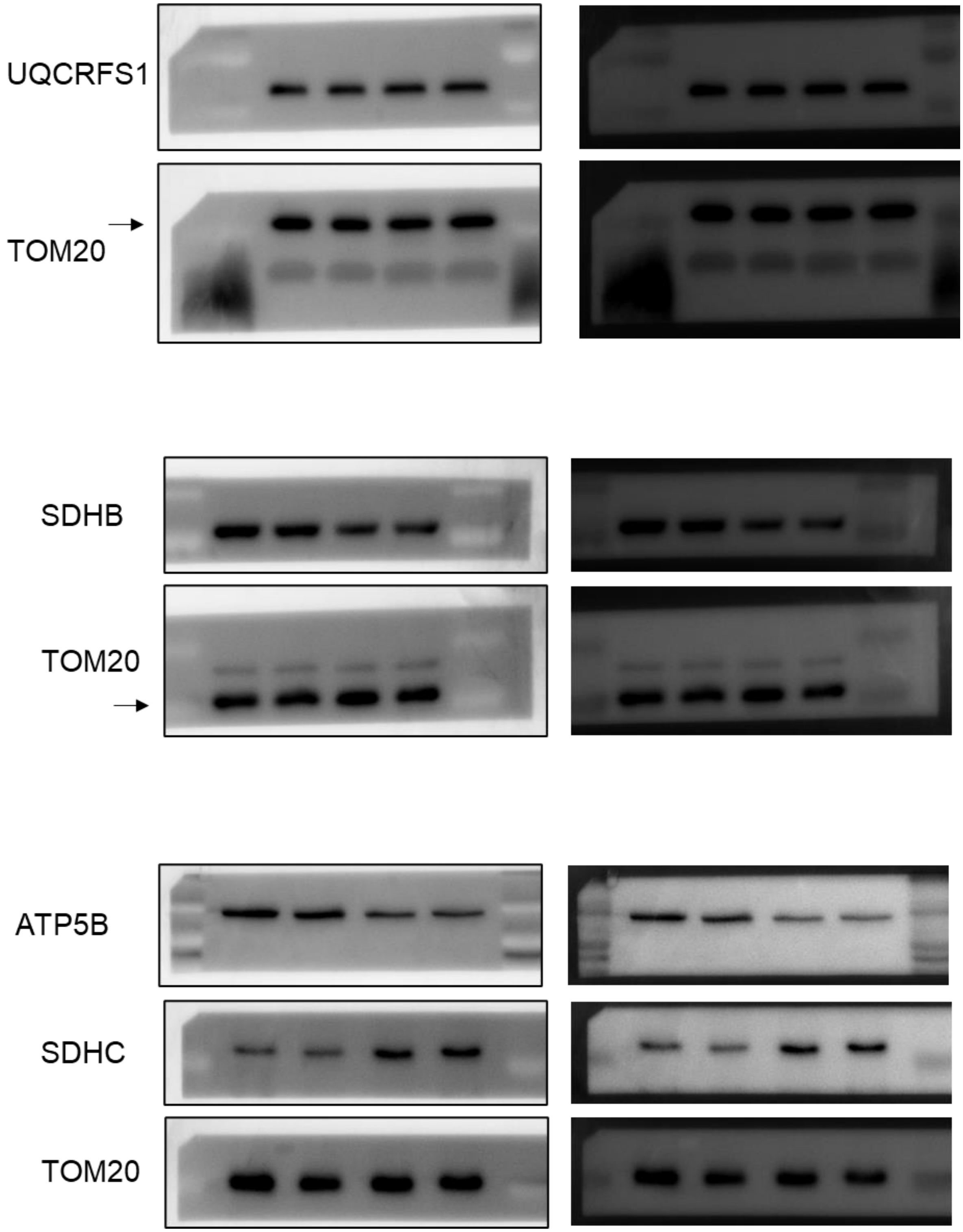
UQCRFS1
TOM20
SDHB
TOM20
ATP5B
SDHC
TOM20

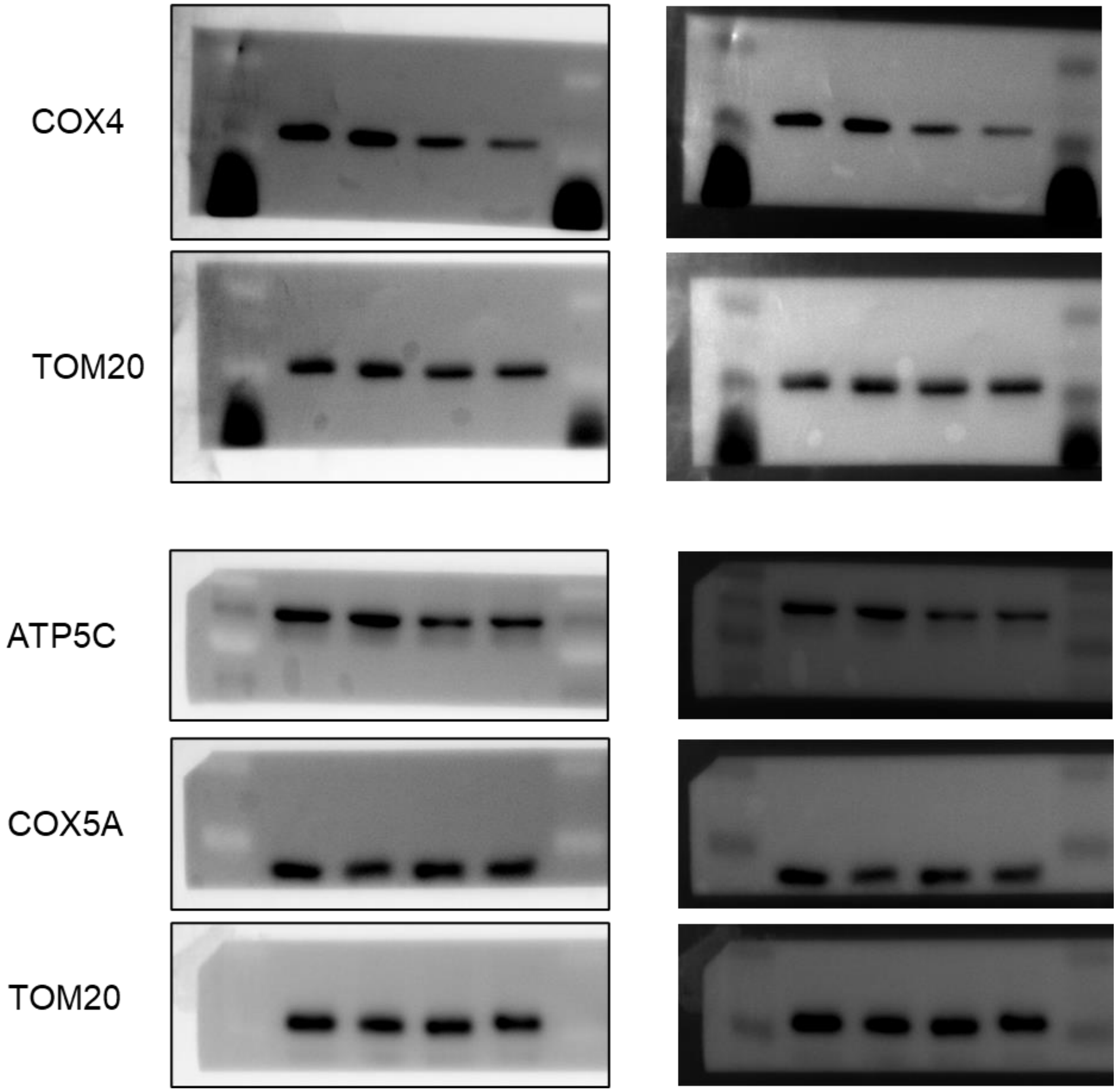

COX4
TOM20
ATP5C
COX5A
TOM20

Full unedited gel for Figure 4D

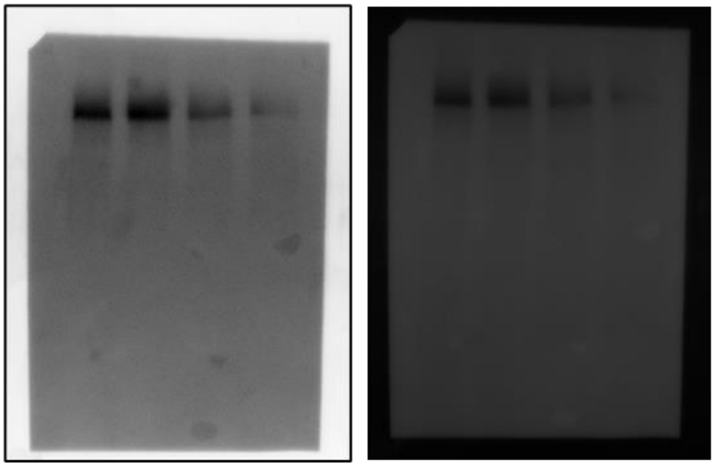

CI: NDUFS1

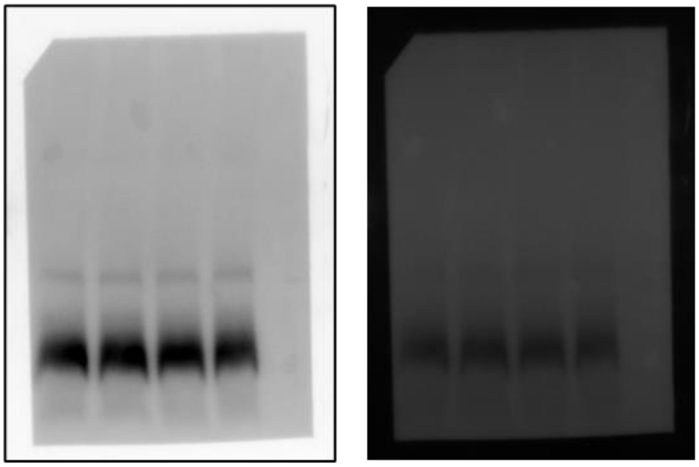

CII: SDHB

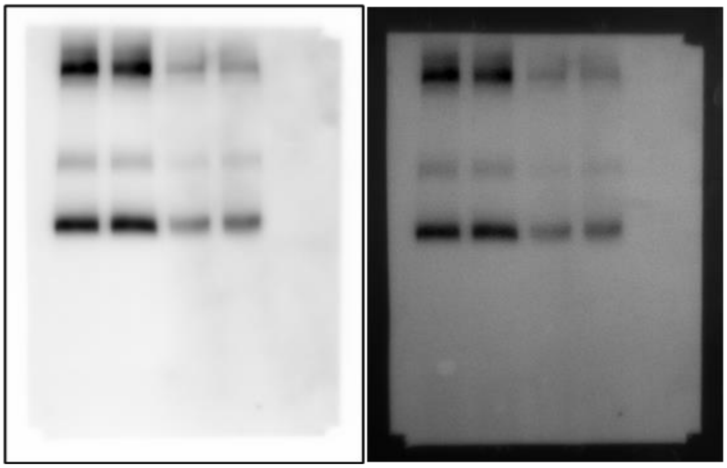

CIII: UQCRC2

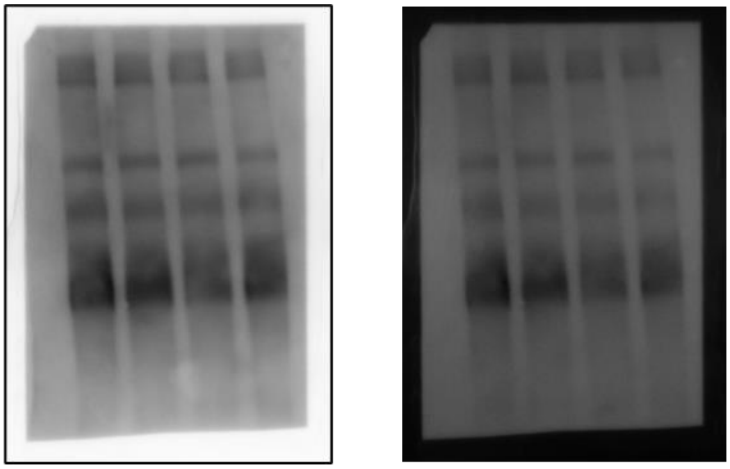

CIV: COX5A

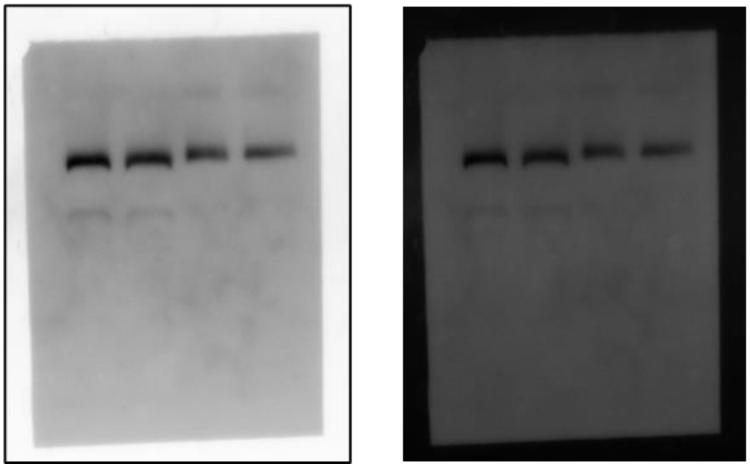

CV: ATP5A

Full unedited gel for Figure 5C

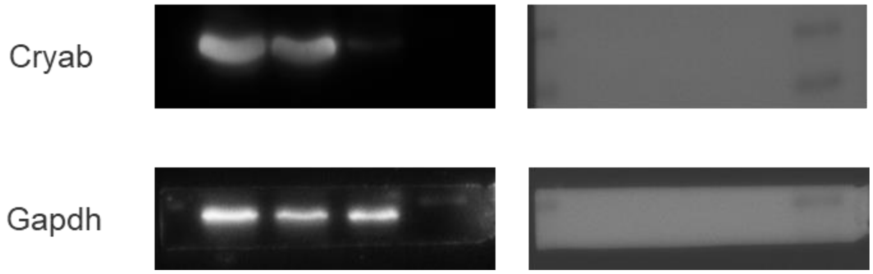

Full unedited gel for Figure 6B

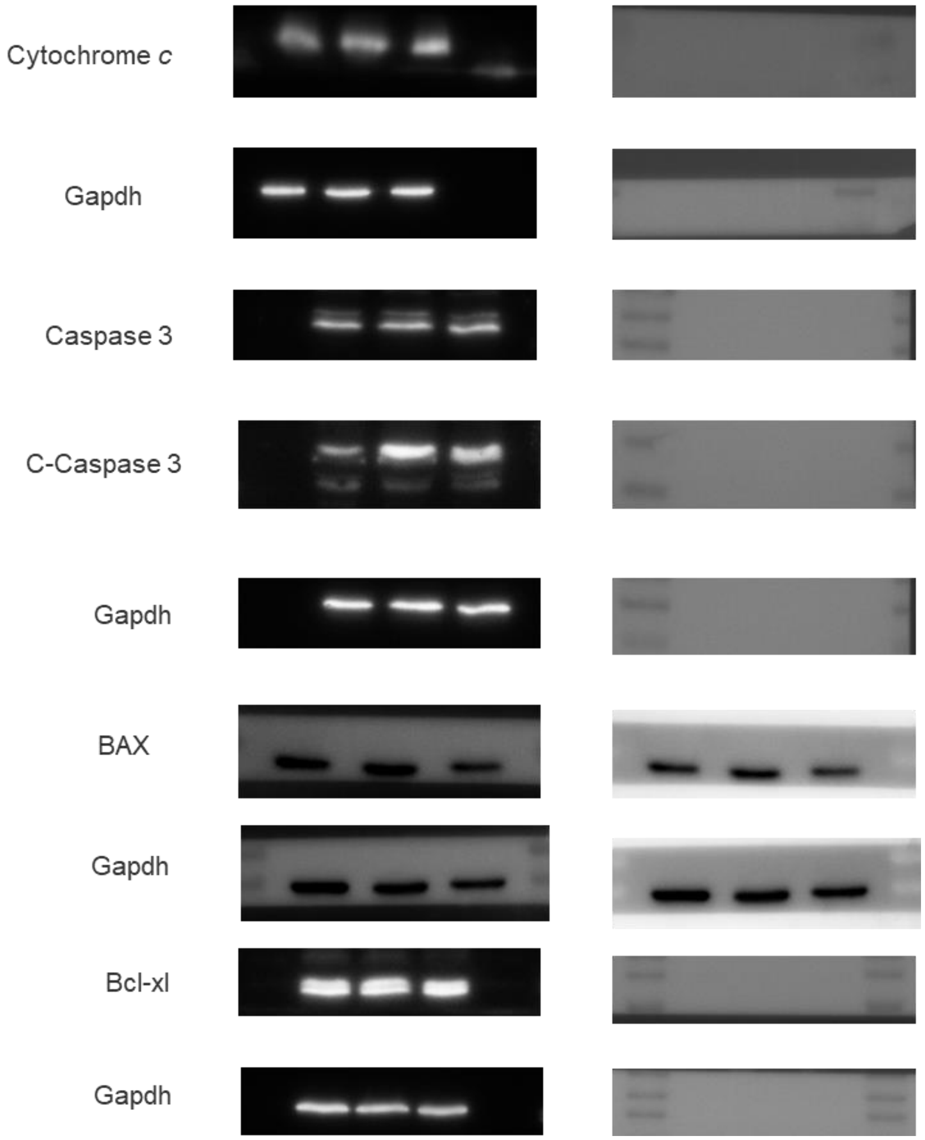

Full unedited gel for Figure 6C

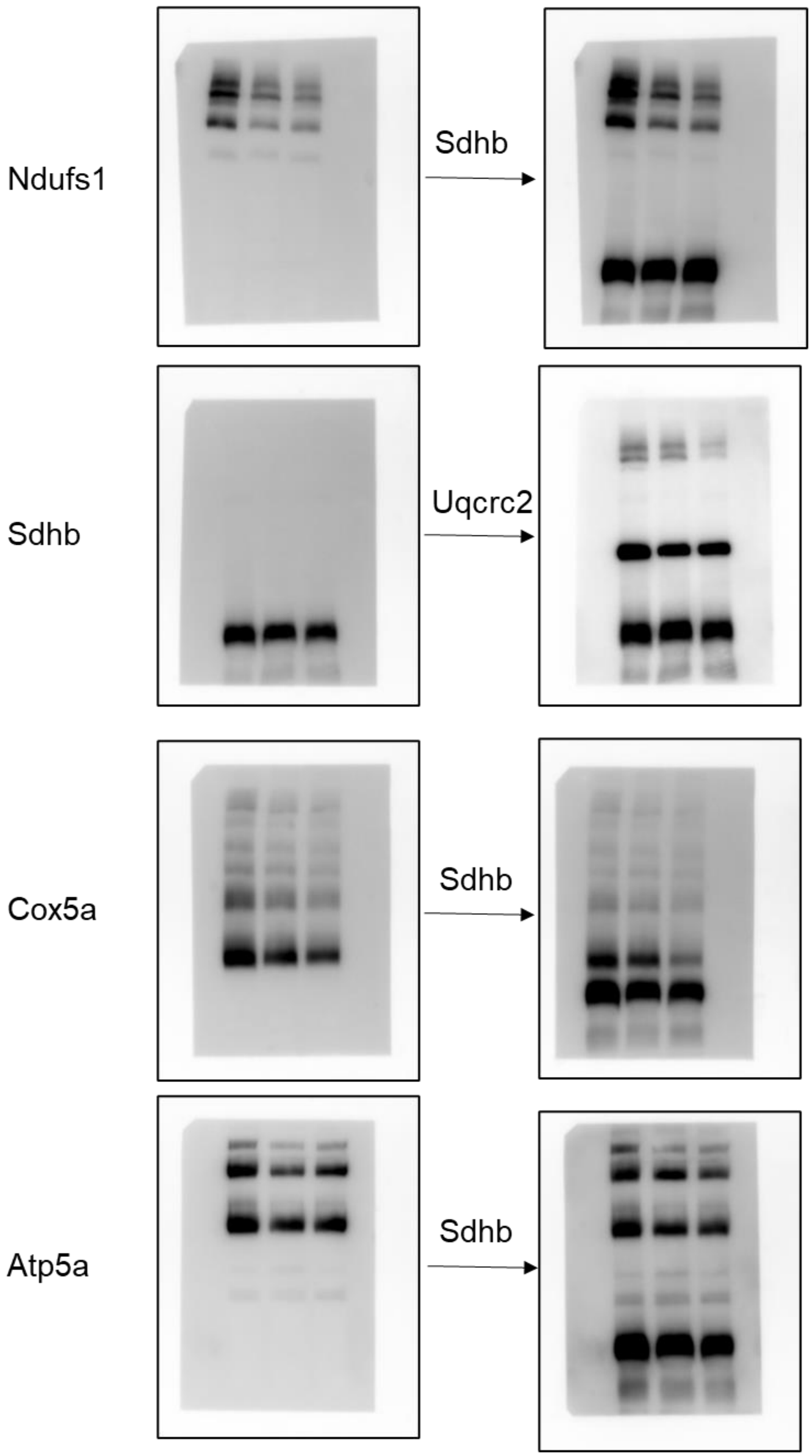

Full unedited gel for Figure 6E

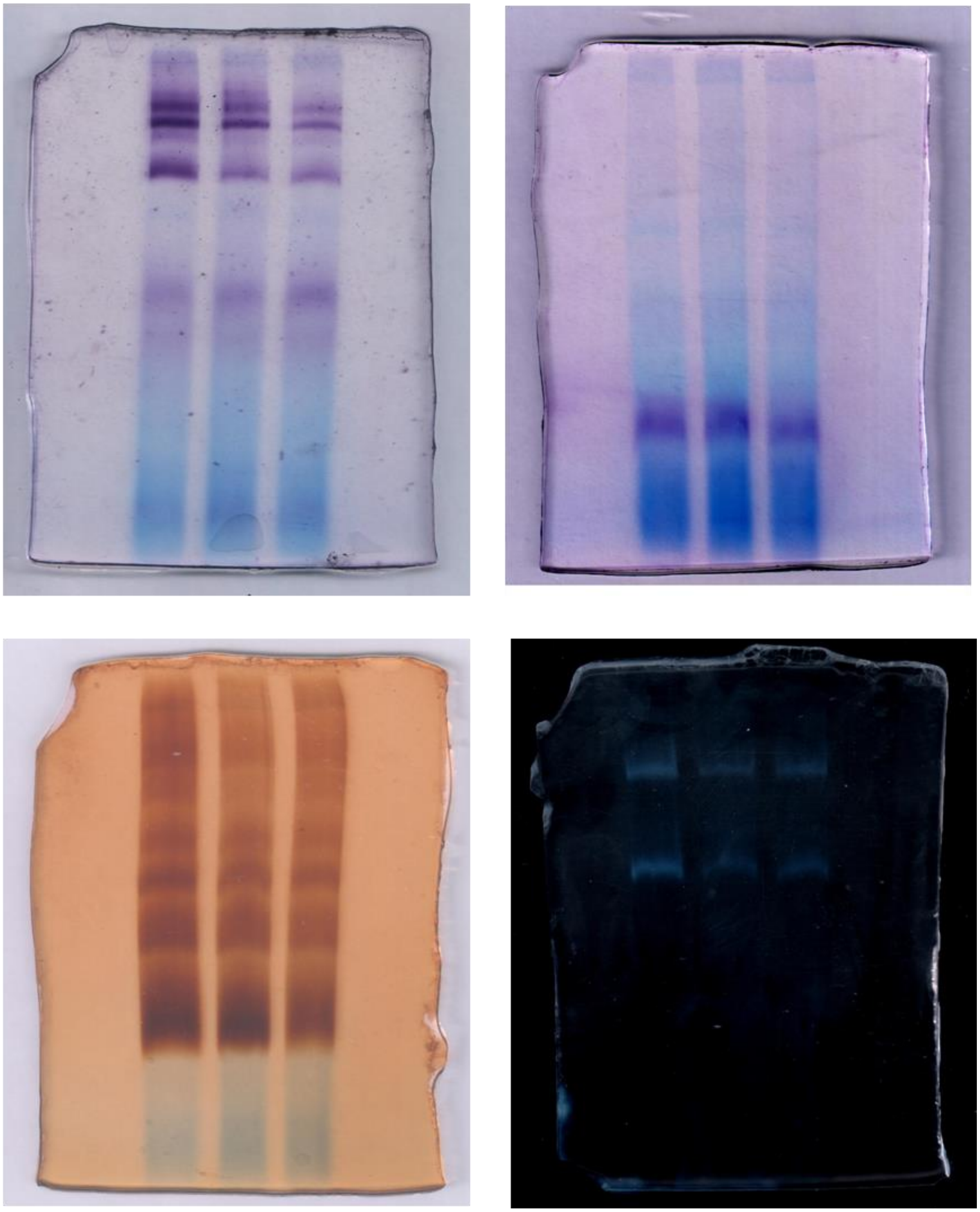

Full unedited gel for Figure S 4A

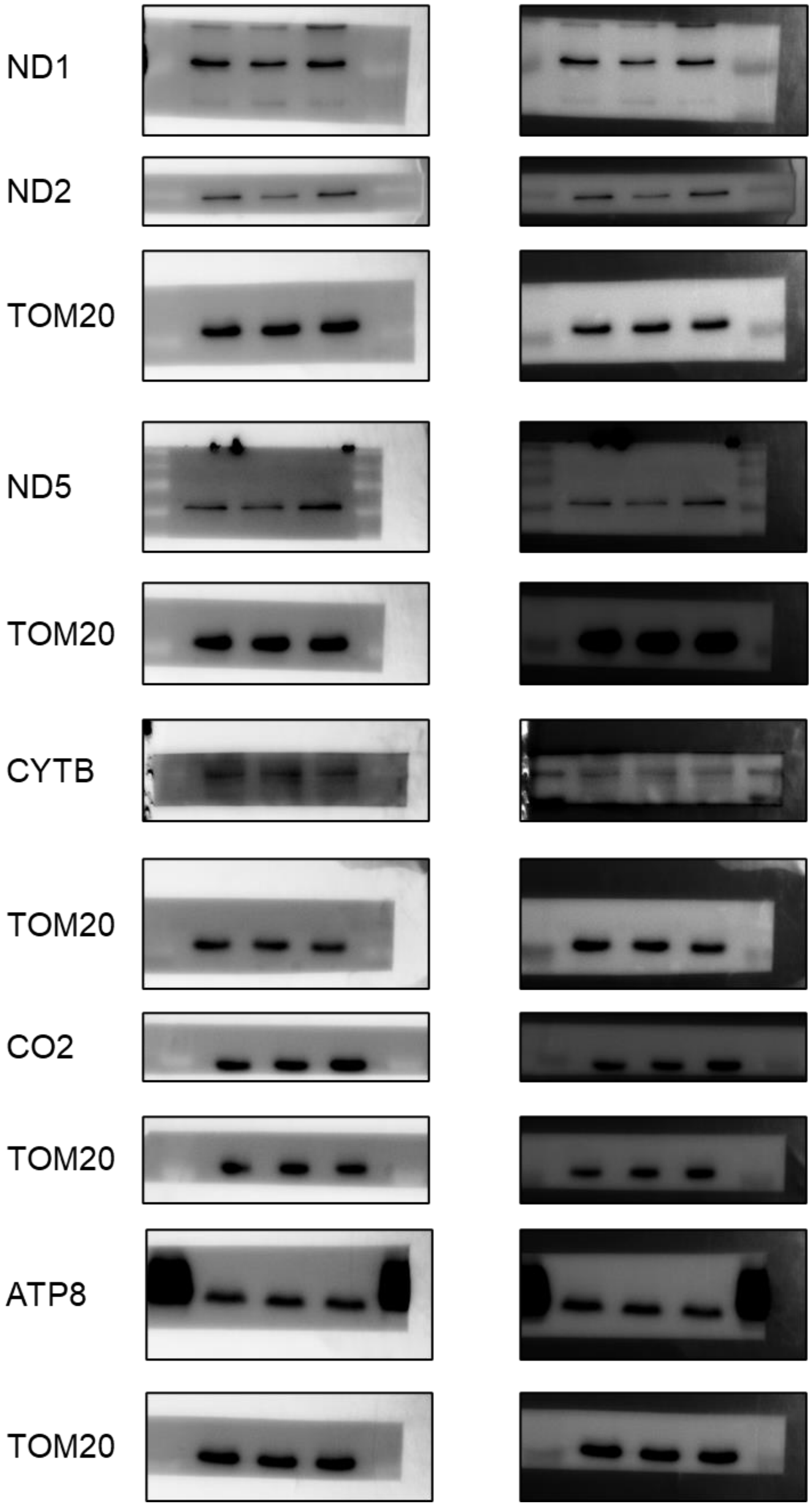

Full unedited gel for Figure S 4C

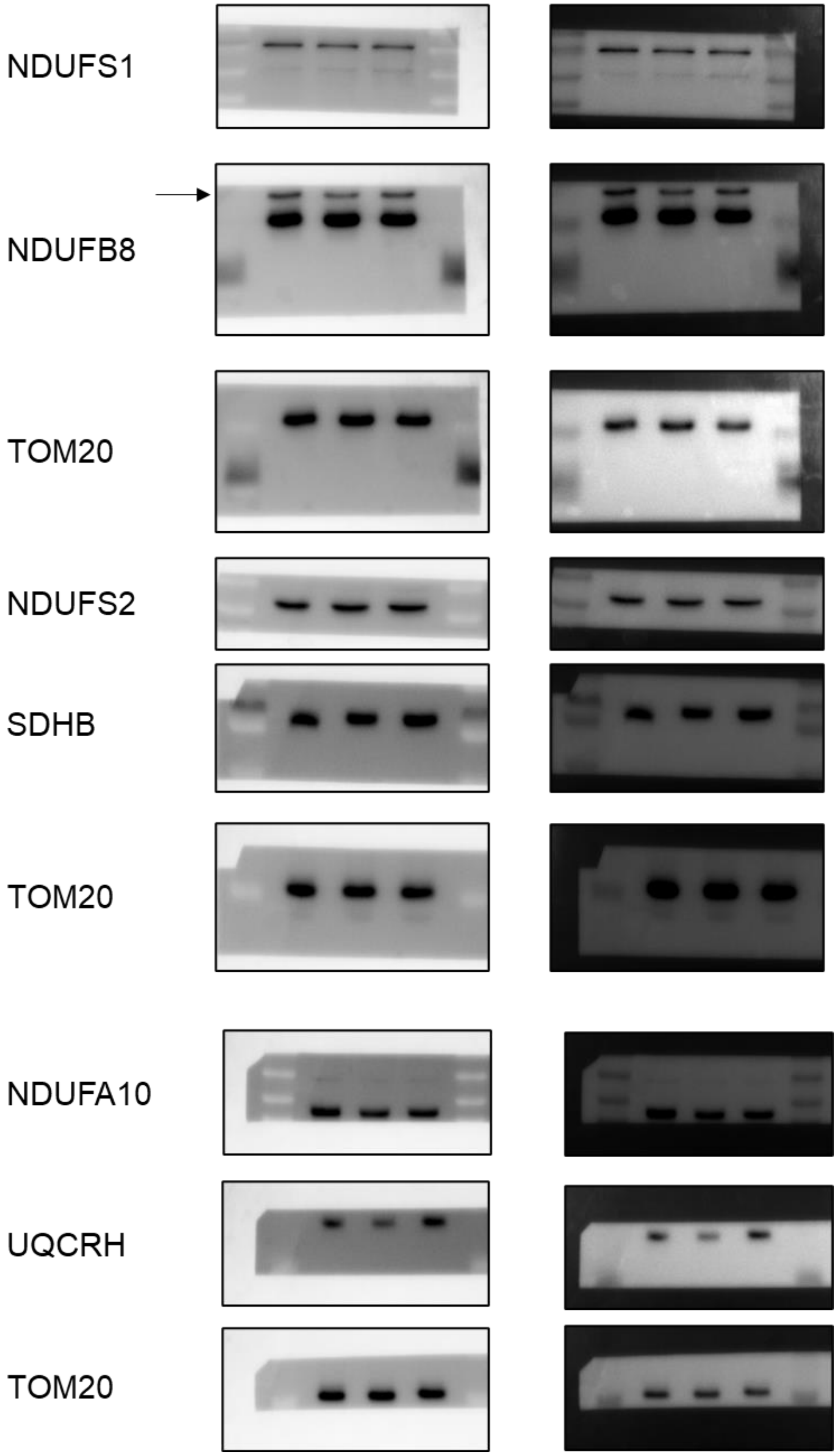

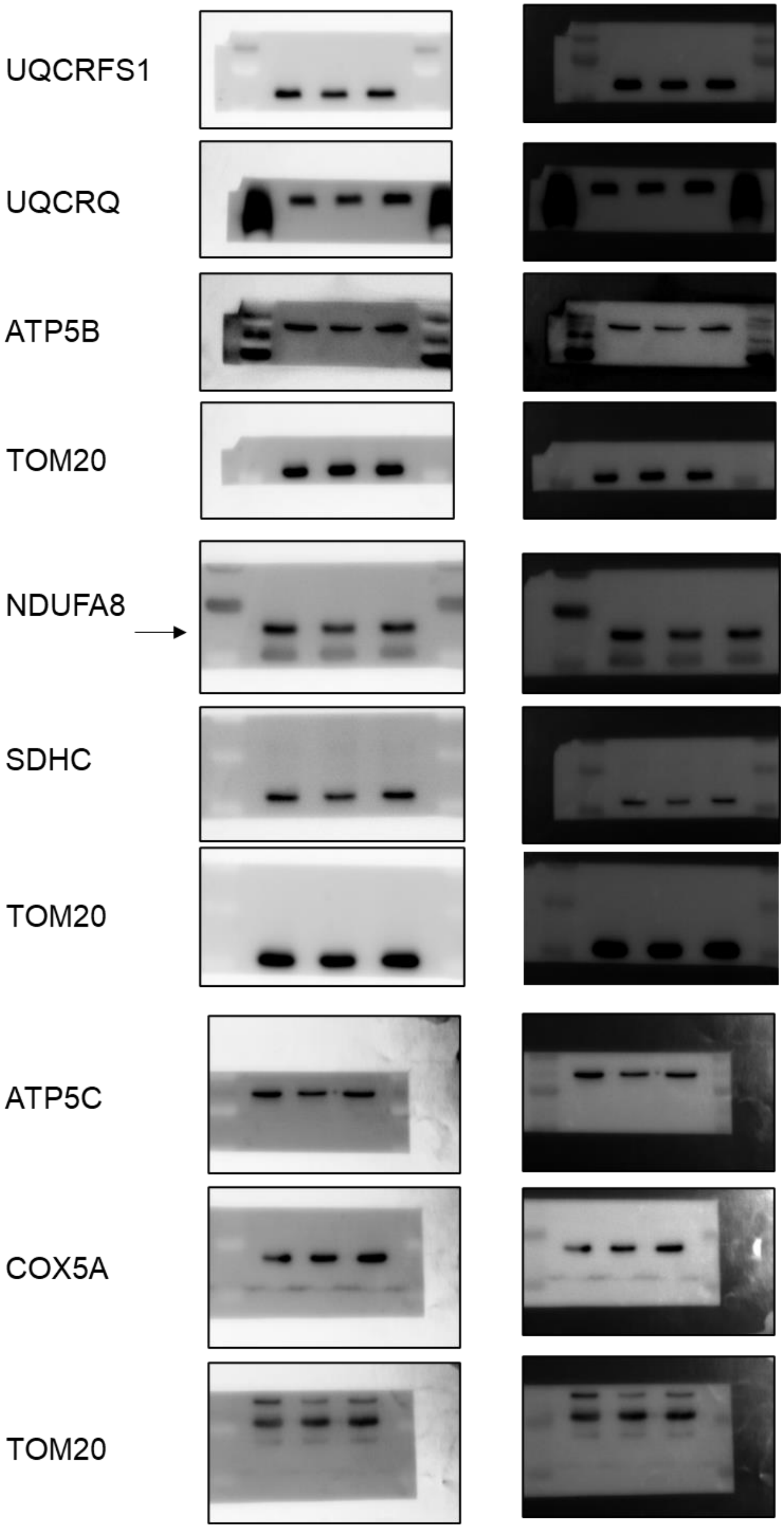
UQCRFS1
UQCRQ
ATP5B
TOM20
NDUFA8
SDHC
TOM20
ATP5C
COX5A
TOM20

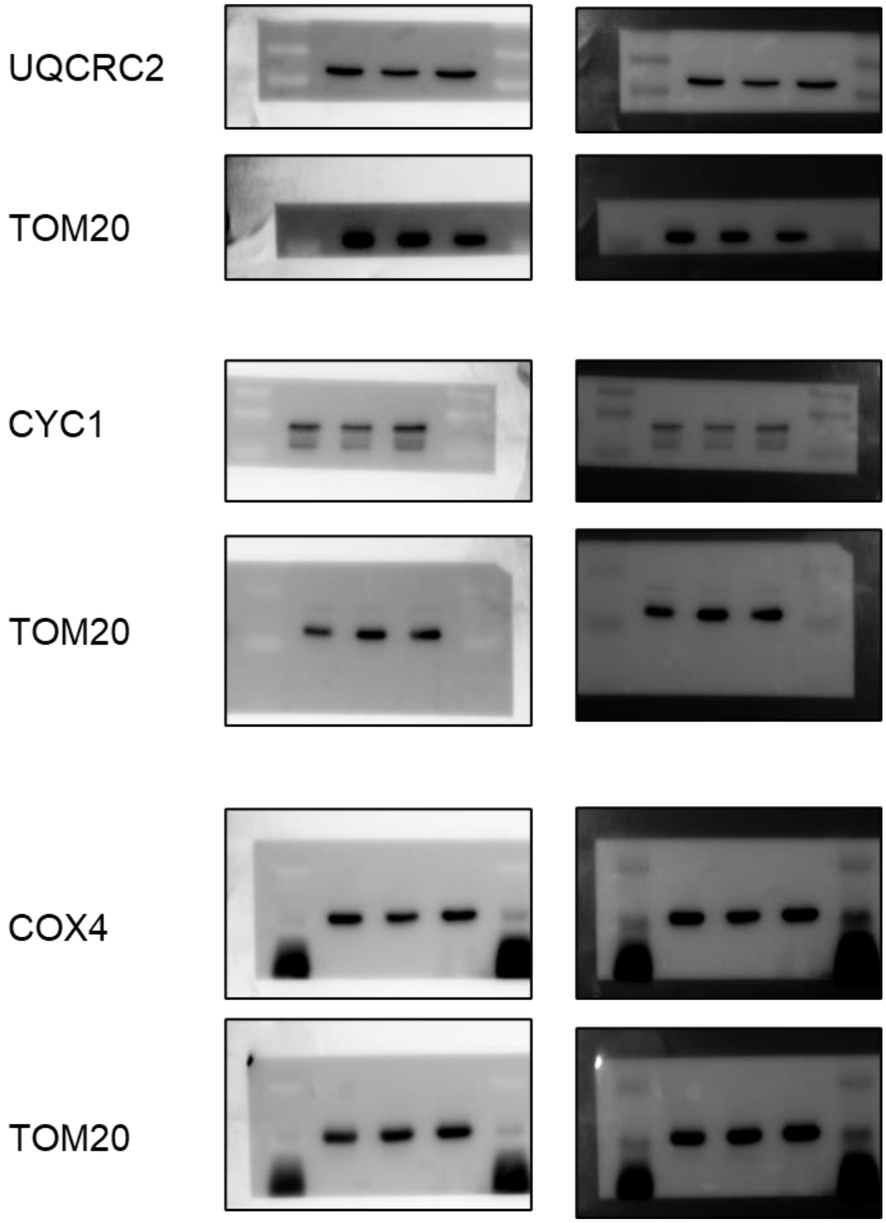
UQCRC2
TOM20
CYC1
TOM20
COX4
TOM20

Full unedited gel for Figure S 5B

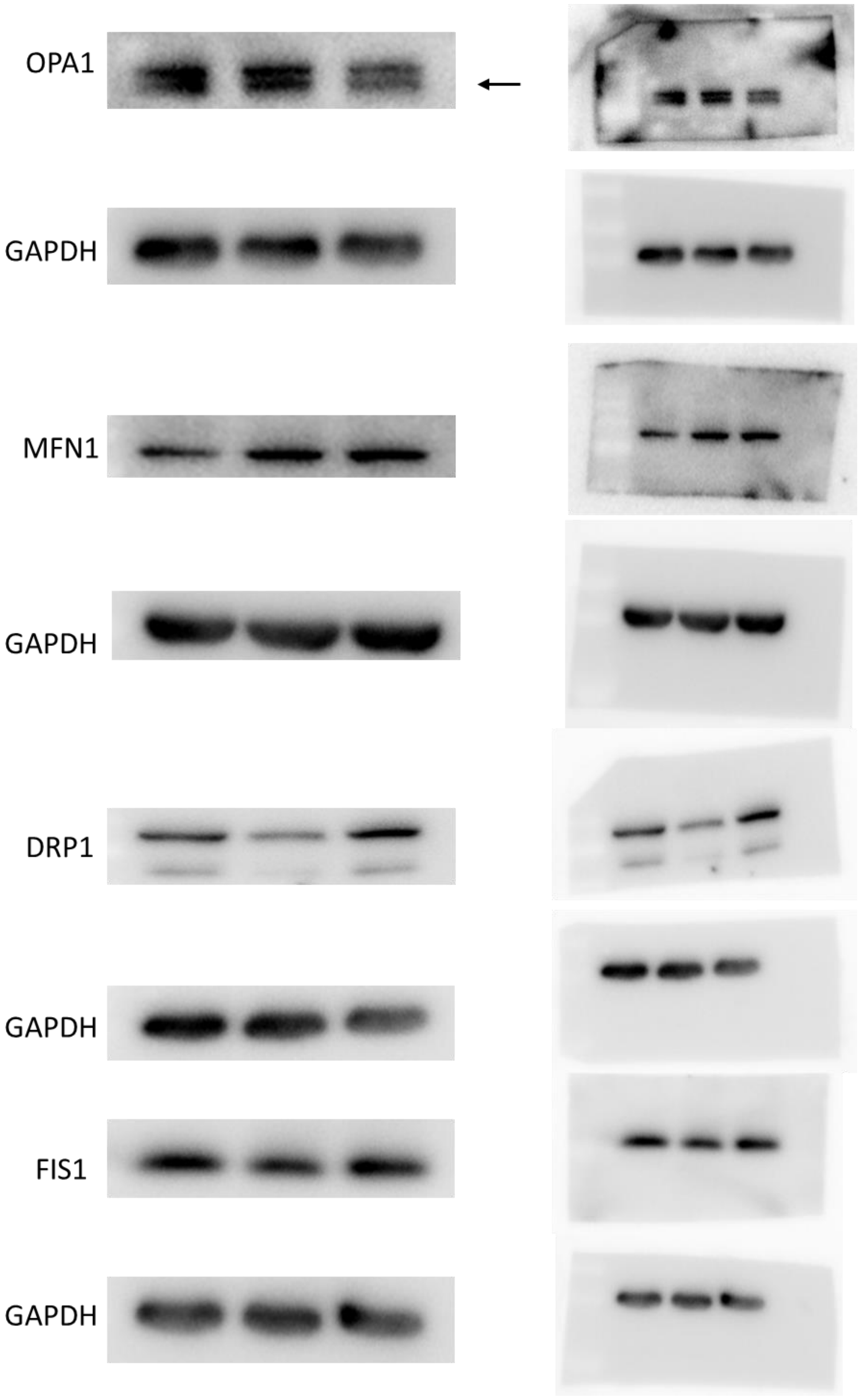

Supplement: Unedited blot and gel images [file jciinsight-10-182209-s281.pdf]
